# *Meso*-Formyl Functionalization Enhances Electrocatalytic Hydrogen Evolution Activity of Nickel(II) Octaethylporphyrin

**DOI:** 10.1021/acs.inorgchem.5c00498

**Published:** 2025-05-27

**Authors:** Tilahun Wubalem Tsega, Danicah T. Agub, Susan D. Arco, Chi-Kwong Chang, Chen-Hsiung Hung

**Affiliations:** † Institute of Chemistry, 38017 Academia Sinica, Nankang, Taipei 115201, Taiwan; ‡ Sustainable Chemical Science and Technology Program, Taiwan International Graduate Program, Academia Sinica, Nankang, Taipei 115201, Taiwan; § Department of Applied Chemistry, National Yang Ming Chiao Tung University, Hsinchu 30010, Taiwan; ∥ Department of Chemistry, College of Natural Sciences, Jimma University, P.O. Box 378, Jimma 251, Ethiopia; ⊥ Institute of Chemistry, 54727 University of the Philippines Diliman, Quezon City 1101, Philippines; # Department of Medicinal and Applied Chemistry, Kaohsiung Medical University, Kaohsiung 807378, Taiwan

## Abstract

Molecular catalysts optimized with functional groups offer promising alternatives to precious metals for hydrogen evolution reactions (HER). This study investigates the electrocatalytic enhancement achieved by aldehyde functionalization at the *meso*-carbon position in 5-formyloctaethylporphyrin-nickel­(II) ([Ni­(OEP-CHO)]). Spectroscopic, cyclic voltammetry (CV), controlled potential electrolysis (CPE), and ultraviolet visible (UV–vis) Spectro electrochemistry reveal that the formyl group modulates the electronic properties of the porphyrin complex. In the absence of a proton source, CV of [Ni­(OEP-CHO)] shows anodically shifted redox couples at −1.35 and −1.71 V vs Fc/Fc^+^, indicating redox activity and the electron-withdrawing nature of the aldehyde group. In 5 mM TFA, [Ni­(OEP-CHO)] exhibits a lower catalytic onset potential, positively shifted by 0.38 V compared to [Ni­(OEP)]. Titration with TFA reveals acid-independent saturation behavior at concentrations above 15 mM, consistent with a proton-shuttling mechanism involving the formyl group. CPE confirms superior HER performance of [Ni­(OEP-CHO)], achieving a Faradaic efficiency of (96.5 ± 3.8)% and a turnover frequency (TOF) of 0.54 ± 0.04 h^–1^ at an overpotential of 128 mV over 3 h. Notably, its position on Tafel plots reflects high catalytic activity at low overpotentials. These findings present a promising strategy for enhancing porphyrin-based molecular catalysts toward efficient hydrogen fuel production.

## Graphical abstract

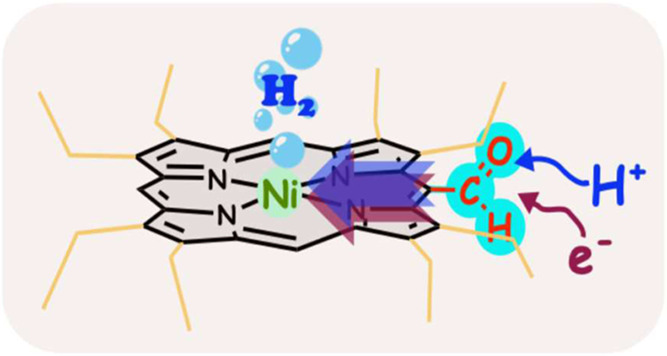

## Introduction

Manipulating molecular architectures by modifying supporting ligands represents a powerful approach to enhancing electrocatalytic performance, positioning molecular catalysts as efficient alternatives to precious metals in energy fuel generation.^1,2^ The hydrogen generation reaction (HER) involves sequential electrochemical redox processes, precise tuning of the energetic profile at a particular step is essential for optimizing catalytic efficiency.^3^ Porphyrinic macrocycles, widely studied as ligands, have undergone various functionalization strategies to enhance their catalytic activity.^4^ For instance, incorporating electron-withdrawing groups can lower the operational potentials required for HER, thereby facilitating electrochemical reduction. However, this modification may impede metal-hydride formation by reducing electron density at the central metal ions.^5^ Conversely, although electron-donating groups facilitate protonation by increasing electron density, they often require higher (more negative) reduction potentials. Recent studies indicate that incorporating nitrogen moieties into the second coordination sphere of porphyrins enhances turnover frequency and promotes proton-coupled electron transfer (PCET), effectively regulating proton stoichiometry during H_2_ evolution.^6–8^ Additionally, hydrogen-bonding groups positioned near the catalytic metal center exert stronger effects compared to those located farther away,^4,9,10^ while polar, charged, or ionizable substituents play a critical role in improving PCET efficiency and the HER activity.^11^ However, despite growing interest in structural tuning, postsynthetic modification strategies remain underutilized in the development of molecular catalysts, offering an untapped avenue for fine-tuning catalytic properties with potentially greater flexibility and efficiency.^12^

Postsynthetic functionalization of porphyrinic macrocycles can be performed at either the *meso*-carbon or β-pyrrolic positions.^13^ However, in metal complexes of octaethylporphyrin (OEP), the substitution of the β-pyrrolic positions with ethyl groups restricts functionalization to the *meso*-positions. Functionalization at *meso*-carbons significantly affects the symmetry and energy levels of the molecular orbitals due to their strong coupling with the π-conjugated system. *Meso*-formylated [Ni­(OEPH–CHO)] has been employed as a precursor for constructing conjugated porphyrin oligomers,^14–16^ but its electrocatalytic properties remain unexplored. Herein, we report the electrocatalytic HER activity of [Ni­(OEP-CHO)], highlighting the dual functional roles of the *meso*-formyl group—its redox-active nature and the proton-shuttling capability of its oxygen atom—in the presence of a strong acid, which collectively enhanced HER. This work exemplifies how *meso*-functionalization can endow porphyrin frameworks with synergistic electronic and proton-relay features, offering a promising design strategy beyond traditional electron-donating or electron-withdrawing substitutions. Our findings reveal that [Ni­(OEP-CHO)] exhibits sustainable current enhancement and lower onset potentials compared to [Ni­(OEP)] in DMF with trifluoroacetic acid (TFA) as the proton source. A turnover frequency of 0.54 ± 0.04 h^–1^ was achieved for using [Ni­(OEP-CHO)] as the electrocatalyst, surpassing the TOF of 0.13 h^–1^ for [Ni­(OEP)] under identical conditions.

## Experimental Section

### Materials and Instruments

All starting materials, including reagent-grade phosphorus oxychloride (POCl_3_), dimethylformamide (DMF), tetra-*n*-butylammonium hexafluorophosphate ([Bu_4_NPF_6_]), sodium acetate (NaOAc), sodium sulfate (Na_2_SO_4_), hexane, chloroform (CHCl_3_), and dichloromethane (CH_2_Cl_2_), were used as received without further purification. Nickel­(II) octaethylporphyrin ([Ni­(OEP)]) were prepared as described in the literature.^17–19^ Characterization was performed using ultraviolet visible (UV–vis) spectroscopy (Agilent 8453 spectrophotometer), ^1^H NMR (Bruker Advance III HD 400 MHz NMR Spectrometer), and IR spectroscopy (Bruker Vertex 70v). High-resolution electrospray ionization mass spectrometry (HR-ESI-MS) was carried out on a Waters Q-TOP Premier equipped with electrospray ionization (ESI) source operating in positive mode. Elemental analysis was carried out using an Elementar Vario El Cube.

### Synthesis of 5-Formyl-2,3,7,8,12,13,17,18-Octaethylporphyrin Nickel­(II), [Ni­(OEP-CHO)]

[Ni­(OEP-CHO)] was synthesized following standard Vilsmeier formylation procedures.^14^ To a magnetically stirred solution of DMF (10.6 mL) in an ice bath, POCl_3_ (12.3 mL, 0.13 mol) was added dropwise under cooling. After complete addition, the mixture was stirred at 0 °C for 15 min, then allowed to warm to room temperature and stirred for an additional 30 min. Separately, a solution of [Ni­(OEP)] (0.25 g, 0.41 mmol) in CH_2_Cl_2_ (800 mL) was prepared and added dropwise to the Vilsmeier reagent over 30 min under magnetic stirring. The resulting reaction mixture was then heated to 50 °C and maintained for 1 h. After cooling to room temperature, the reaction was quenched by careful addition of saturated solution of NaOAc_(aq)_ and stirred for an additional 2 h. (*Caution: Do not add aqueous solutions to hot CH*_2_ *Cl*_2_, *as this may lead to pressure buildup and overflow.*) The organic phase was separated, washed with distilled water, dried over anhydrous Na_2_SO_4_, filtered, and concentrated under reduced pressure. The crude product was purified by column chromatography using a 50:50 CHCl_3_/hexane eluent to afford [Ni­(OEP-CHO)] as a red solid (202.15 mg, 77% yield). Crystals suitable for single-crystal X-ray diffraction were obtained by slow diffusion of hexane into a CH_2_Cl_2_ solution of [Ni­(OEP-CHO)] over 2 days.

#### Characterization Data

HR-ESI-MS: *m*/*z* [M]^+^ calcd: 618.2863; Found: 618.2856; ^1^H NMR (CDCl_3_, 400 MHz): δ 11.81 (s, 1H), 9.25 (s, 1H), 9.21 (s, 2H), 3.67–3.63 (dd, *J* = 17.7 and 7.6 Hz, 16H), 1.76–1.57 (m, 24H). UV–vis (CH_2_Cl_2_): λ­[nm] (log ε [M^–1^ cm^–1^]): 402 (3.87), 420 (3.84), 524 (2.61), 560 (2.86). Elemental Analysis: Calcd for C_37_H_44_N_4_NiO·0.1CHCl_3_: C, 70.57; H, 7.04; N, 8.87; O, 2.53. Found: C, 70.52; H, 7.18; N, 8.58; O, 2.79. IR­(cm^–1^): 2955, 2927, 2865 (*v*(C–H)), 1650 (*v*(C=O)), 1440 (*v*(C=N)).

### Crystal Structure Determination of [Ni­(OEP-CHO)]

Single-crystal X-ray diffraction data for [Ni­(OEP-CHO)] were collected at 100 K using a Bruker D8 Venture diffractometer equipped with a PHOTON III detector and Cu Kα radiation (λ = 1.54178 Å). A suitable crystal was mounted on a goniometer and cooled to the data collection temperature under a nitrogen gas stream. Data reduction was performed using the APEX3 software suite, and a multiscan absorption correction was applied. The structure was solved by direct methods and refined by full-matrix least-squares on *F* ^2^ using SHELXL. Non-hydrogen atoms were refined anisotropically, and hydrogen atoms were placed in calculated positions and refined using a riding model. The dark-red needle crystal of dimensions 0.513 × 0.079 × 0.033 mm^3^ crystallized in the triclinic system, space group *P*1̅, with unit cell parameters *a* = 4.8095(3) Å, *b* = 12.8139(9) Å, *c* = 13.4104(9) Å, α = 68.001(3)°, β = 89.532(3)°, γ = 84.498(3)°, and *V* = 762.41(9) Å^3^. Crystallographic data have been deposited with the Cambridge Crystallographic Data Centre under deposition number CCDC 2440554.

### Electrochemical and Catalytic Properties

The electrocatalytic behavior of the samples was investigated using a CHI72 electrochemical analyzer equipped with a three-electrode system. In organic medium, a three-electrode setup was employed with an Ag wire as the pseudoreference electrode, a glassy carbon electrode (GCE) as the working electrode, and a platinum wire as the counter electrode. Prior to each measurement, the GCE was polished and pretreated with fresh 0.1 M [Bu_4_N]­PF_6_ supporting electrolyte. IR compensation was performed before cyclic voltammetry (CV) or linear sweep voltammetry (LSV) measurements. All potentials are referenced against the Fc/Fc^+^ redox couple. The solution was saturated with N_2_ before measurements and all experiments were carried out in a tightly sealed electrochemical vessel at room temperature with a scan rate of 0.1 V/s. The electrocatalytic activity was assessed based on the current intensity obtained from CV and LSV measurements, as well as the charge accumulation during controlled potential electrolysis (CPE) in the presence of TFA as the proton source. Controlled experiments using a blank solution and [Ni­(OEP)] were conducted to determine background contributions. To evaluate surface adsorption, the working electrode was rinsed thoroughly with water after CV or CPE, then reintroduced into a catalyst-free supporting electrolyte. The absence of catalytic current confirmed that no catalytic species remained adsorbed on the electrode surface. Hydrogen production was verified using gas chromatograph (Figure S12c) and quantify though an H_2_ gas calibration curve. The Faradaic efficiencies were then calculated as the ratio of electron consumed in H_2_ production to the total accumulated charge during electrolysis.

### Computational Details

All quantum chemical calculations were performed using the ORCA 6.0.1 program package.^20,21^ The initial geometry of the [Ni­(OEP–CHO)] complex was derived from the single-crystal structure of octaethyl-5-formylporphyrin (CCDC: EBVFPC10),^22^ which features the characteristic four-up–four-down ethyl orientation and saddle distortion typical of Ni­(II) porphyrins. The structure was modified in Avogadro by replacing the central Cu with Ni and removing the substituent at the alternate *meso* position. Hydrogen atoms, including those at the formyl oxygen or coordinated to Ni, were added and refined via geometry optimization. All structures were optimized using ORCA 6.0. Protonation and reduction states were modeled by adjusting the total charge and spin multiplicity, with both low- and high-spin states examined for Ni­(III) species. The computational procedure comprised three main steps. First, a geometry optimization was performed at the Restricted Hartree–Fock (RHF) level using the def2-SVP basis set. Next, a vibrational frequency analysis at the HF/def2-SVP level confirmed the absence of imaginary frequencies, ensuring that the optimized structure corresponded to a true local minimum. Finally, a single-point energy calculation was conducted at the hybrid DFT PBE0 level, employing the def2-TZVP basis set and the def2/J auxiliary basis set.

## Results and Discussion

### Characterization and Electrochemistry of [Ni­(OEP-CHO)]

The compound, [Ni­(OEP-CHO)] was synthesized according to reported procedures.^16,23^ The as-prepared [Ni­(OEP-CHO)] was subjected to a range of physicochemical characterizations and compared with reported data. In CH_2_Cl_2_, its UV–vis spectrum displayed a red-shifted, split *Soret* band at 402 and 420 nm, along with broadened Q bands extending from 524 to 680 nm, relative to its precursor [Ni­(OEP)] (Figure S5A **)**. A distinctive resonance at 11.81 ppm in the ^1^H NMR spectrum corresponds to the *meso*-formyl proton, while the remaining three *meso*-protons appear as two separate signals with a 1:2 integration ratio at 9.34 and 9.31 ppm, confirms the identity and purity of [Ni­(OEP-CHO)] (Figure S6).^16^ The IR spectrum exhibits a strong band at 1650 cm^–1^, consistent with C=O stretching, along with additional bands at 2927 and 2865 cm^–1^ attributable to aldehyde C–H stretching modes (Figure S5B).

Single-crystal X-ray diffraction analysis revealed that [Ni­(OEP-CHO)] crystallizes in the triclinic space group *P*1̅, with the nickel atom situated on an inversion center. Consequently, only half of the molecule is symmetry-independent, and the remainder is generated by symmetry operations. As shown in Figure 1, a formyl substituent is clearly resolved in the crystal structure with a C=O bond length of 1.199(7) Å. The average Ni–N bond length is 1.965(2) Å. The porphyrin macrocycle, defined by 24 core atoms, is essentially planar, exhibiting an averaged deviation of 0.029 Å for the mean plane. The formyl group is tilted by 3.16 ° from the normal to the porphyrin plane.

**Figure 1:**
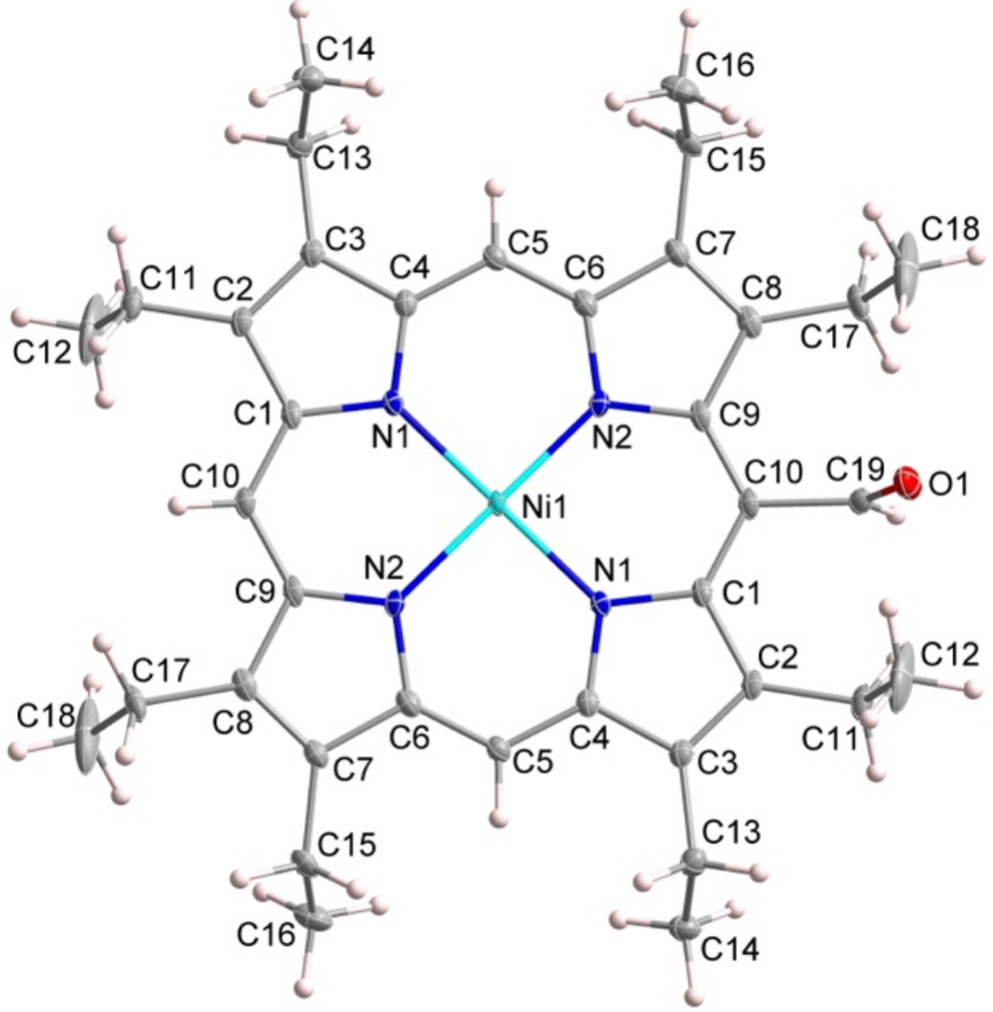
ORTEP diagram of [Ni­(OEP-CHO)] with thermal ellipsoids drawn at the 30% probability level.

The electrochemical properties of [Ni­(OEP-CHO)] were investigated by cyclic voltammetry in 0.1 M [Bu_4_N]­PF_6_/DMF solution. As shown in Figure 2A, [Ni­(OEP-CHO)] exhibits a reversible redox couple at −1.35 V and a less reversible couple at −1.71 V. These reduction potentials are anodically shifted relative to the single redox couple of [Ni­(OEP)], centered at −1.82 V vs Fc/Fc^+^ (Figure 2A), highlighting the electron-withdrawing nature of the formyl group.^24^

**Figure 2:**
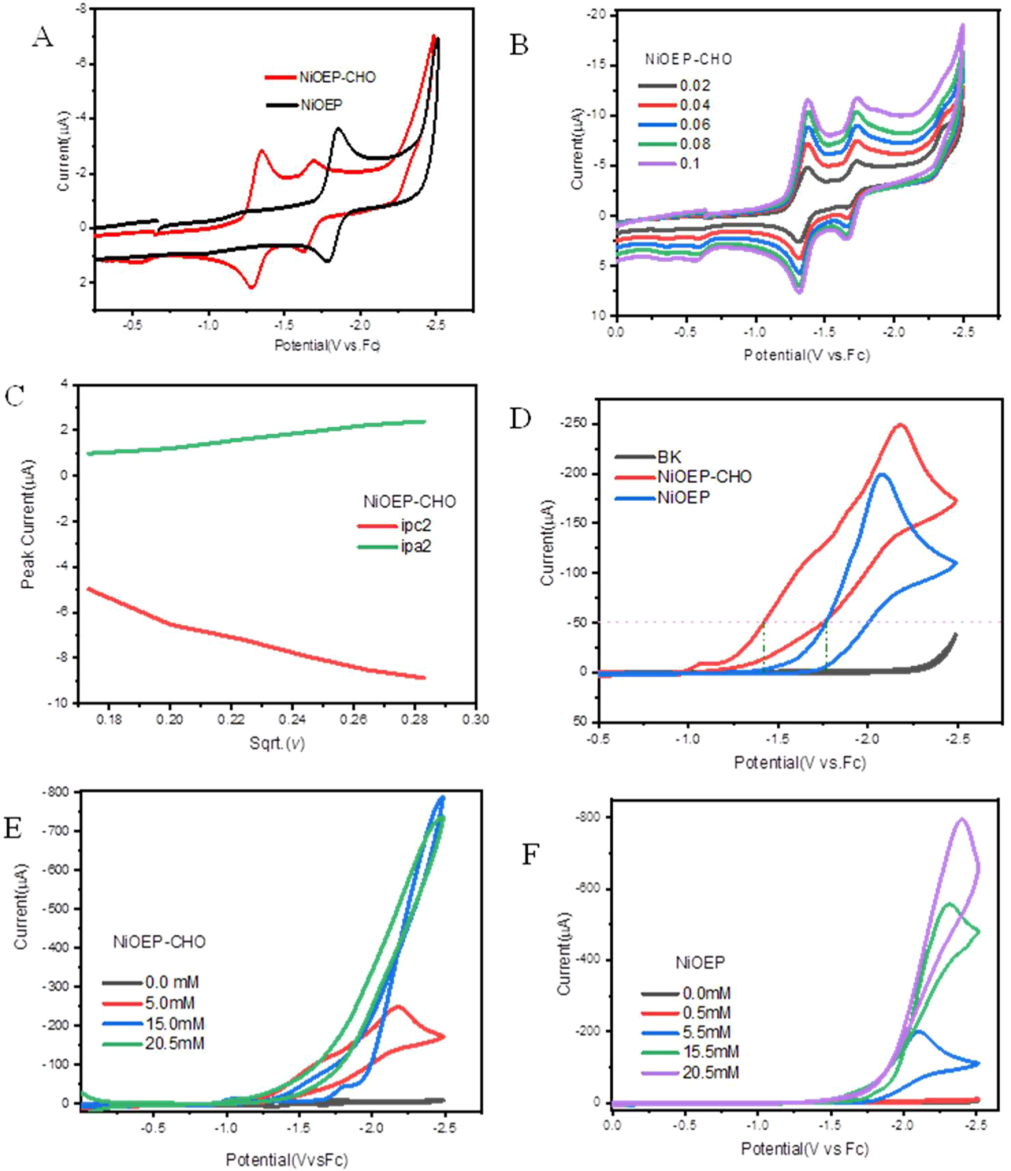
(A) Cyclic voltammograms of [Ni­(OEP-CHO)] (red) and [Ni­(OEP)] (black) in 0.1 M [Bu_4_N]­PF_6_/DMF. (B) Cyclic voltammograms of [Ni­(OEP-CHO)] at various scan rates. (C) Plot of peak current (*i*_p_) versus square root of scan rate (ν^1/2^) for [Ni­(OEP-CHO)]. (D) Cyclic voltammograms of 0.5 mM [Ni­(OEP-CHO)] with 5 mM TFA (red) and 0.5 mM [Ni­(OEP)] with 5 mM TFA (blue). (E) Cyclic voltammograms of 0.5 mM [Ni­(OEP-CHO)] with increasing TFA concentrations: 0 (black), 5 (red), 15 (blue), and 20.5 mM (green). (F) Cyclic voltammograms of 0.5 mM [Ni­(OEP)] with TFA concentrations ranging from 0 (black) to 20.5 mM (purple).

The reduction couple of [Ni­(OEP)] has been primarily attributed to a ligand-based process, generating a Ni^2+^ complex with an OEP anionic radical, [Ni^2+^(OEP^•^)]^−^.^25^ However, an alternative interpretation suggested that the first reduction may be nickel-centered.^26^ Due to the mixing of metal and porphyrin molecular orbitals, redox events in such systems are delocalized, complicating the assignment of a specific redox couple to either the metal center or the ligand.^27^ In related systems such as nickel-tetrakis­(pentafluorophenyl)­porphyrin (NiTFPP), the first redox process has been assigned to a metal-centered reduction. Notably, in a thin-layer spectroelectrochemical setup, UV–vis spectra of NiTFPP exhibited a red-shifted, less intense Soret band, along with the emergence of new absorption bands at 358 and 608 nm upon electrolysis at −1.3 V in CH_3_CN.^24^

The key structural difference between [Ni­(OEP-CHO)] and [Ni­(OEP)] is the presence of the formyl group. While [Ni­(OEP)] exhibits a single redox wave, [Ni­(OEP-CHO)] displays two distinct redox couples. The second redox event in [Ni­(OEP-CHO)] is likely due to the formyl substituent, which may participate in electron or proton shuttling during the redox process. Additionally, [Ni­(OEP-CHO)] shows a spectral pattern to NiTFPP upon electrolysis at −1.12 V (−1.76 V vs Fc^+^/Fc) in 0.1 M [Bu_4_N]­PF_6_/DMF using Pt-mesh working electrode, suggesting a nickel-centered first reduction. Variable scan rate (ν) studies confirmed that all redox events are diffusion-controlled, as evidenced by the linear relationship between peak currents and ν^1/2^ (Figure 2C for [Ni­(OEP-CHO)], Figure S1B for [Ni­(OEP)]).

### Electrocatalytic Activities

To evaluate the influence of the *meso*-formyl group on the electrocatalytic behaviors of [Ni­(OEP-CHO)], we analyzed catalytic current, onset potential, and charge accumulation under fixed concentration of TFA. Upon TFA addition, both [Ni­(OEP)] and [Ni­(OEP-CHO)] exhibited significantly enhanced catalytic currents and positive shifts in onset potential compared to their acid-free responses. Notably, [Ni­(OEP-CHO)] showed a higher catalytic current than [Ni­(OEP)] at moderate acid concentrations. As shown in Figure 2D, the introduction of 5 mM TFA (p*K*_a_ = 6.1 in DMF)^28^ to [Ni­(OEP-CHO)] induced catalytic current onset at −1.42 V, with the current exceeding 50 μA. This onset potential is anodically shifted by 381 mV relative to that of [Ni­(OEP)], measured at a comparable threshold current with 5.5 mM TFA. The substantial anodic shift is attributed to the electron-withdrawing formyl group, which increases electron-deficient at the porphyrin core and facilitates proton-coupled electron transfer during hydrogen evolution.^24^

With increasing TFA concentrations from 0.5 to 10.5 M, [Ni­(OEP-CHO)] exhibit a progressively rise in catalytic current (Figure S1C). However, at concentrations above 15 mM, a decline in catalytic current is observed (Figure 2E). At 20.5 mM TFA, the catalytic current enhancement ratio (*i*_c_/*i*_p_ = 74.10 at *E*_p_ = −2.54 V) decreased relative to the value at 15 mM (Table S2). This attenuation is likely due to saturation of proton accessibility via the formyl group, which may act as both a proton acceptor and an intramolecular proton relay under acid conditions.^29^

Interestingly, [Ni­(OEP)] exhibits a markedly higher *i*_c_/*i*_p_ ratio (218.5 at −2.40 V) under the same conditions. This unexpectedly high value, despite lower catalytic currents, can be rationalized by its substantially lower cathodic peak current in the absence of acid (*i*_p_ = 3.652 μA), approximately one-third that of [Ni­(OEP-CHO)] (*i*_p_ = 9.944 μA; Table S1). Furthermore, at 20.5 mM TFA, both the onset and half-wave potentials of [Ni­(OEP-CHO)] remain more anodic than those of [Ni­(OEP)], reinforcing the role of the formyl group in lowering the energy barrier and enhancing the thermodynamic favorability for hydrogen evolution. Importantly, control experiments confirm that direct TFA reduction at the glassy carbon electrode is negligible under these conditions (Figures 3D and S2), indicating that the observed catalytic activity is predominately attributable to [Ni­(OEP-CHO)].

**Figure 3:**
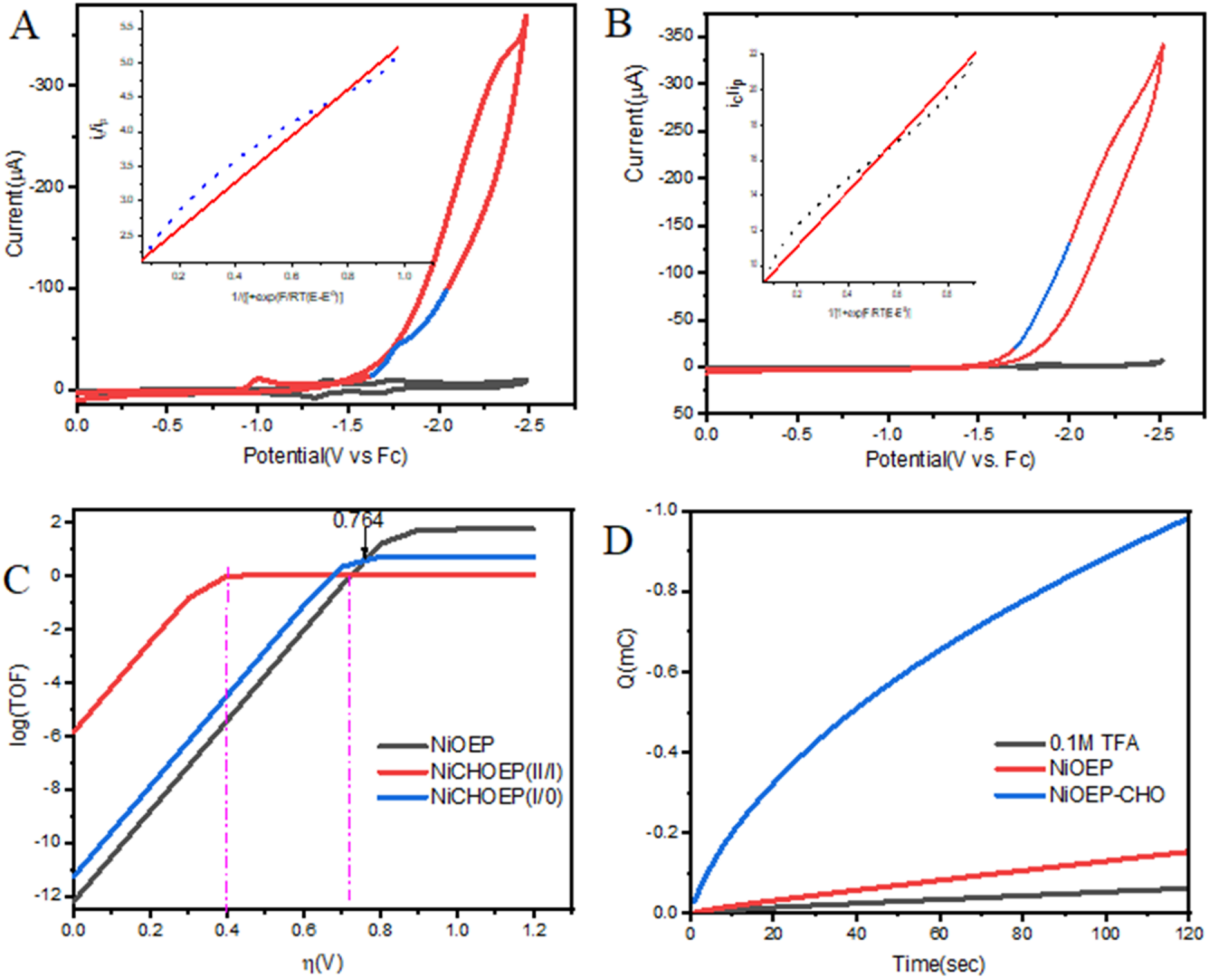
(A) Cyclic voltammograms of [Ni­(OEP-CHO)] in the absence (black) and presence (red) of 100 mM TFA, with the foot-of-the-wave analysis (FOWA) region highlighted (blue); curve fitting shown in the inset. (B) CV and FOWA analysis of [Ni­(OEP)] in the presence of 100 mM TFA. (C) Catalytic Tafel plots for [Ni­(OEP)] (black) and [Ni­(OEP-CHO)] corresponding to the first (blue) and second (red) reduction waves. (D) Controlled potential electrolysis (CPE) traces at [Cat.] = 0.5 mM, [TFA] = 100 mM, and scan rate = 0.1 V s^–1^ for TFA only (black), [Ni­(OEP)] + TFA (red), and [Ni­(OEP-CHO)] + TFA (blue).

Controlled potential electrolysis (CPE) experiments were conducted to validate the HER performance of [Ni­(OEP-CHO)].^30^ At an applied potential of −0.8 V (vs SHE) in 0.1 M TFA, significant charge accumulation was observed after 2 min for [Ni­(OEP-CHO)] (Figure 3D), consistent with the catalytic activity trend shown in the Tafel plot (Figure 3C). Notably, [Ni­(OEP-CHO)] outperforms [Ni­(OEP)] at applied overpotentials below 600 mV. Over a 3 h CPE run, [Ni­(OEP-CHO)] accumulated 1.61 ± 0.08C of charge, yielding a Faradaic efficiency (FE) of (96.5 ± 3.8)% for H_2_ production at an overpotential of 128 mV. In comparison, [Ni­(OEP)] accumulated only 0.185C, corresponding to an FE of 78% under identical conditions. These results clearly demonstrate that formylation enhances charge accumulation, reduces the operational overpotential, and improves HER selectivity and efficiency.

In the presence of acetic acid (10 mM) as the proton source, the catalytic current profiles differ markedly from those observed with TFA. For [Ni­(OEP-CHO)], the voltammograms display consistently lower catalytic current enhancement compared to those in TFA, accompanied by irreversible reduction waves at −1.31, −1.96 and −2.22 V (vs Fc/Fc^+^) (Figure 4A). In contrast, the voltammogram of [Ni­(OEP)] in acetic acid closely resembles its behavior in TFA, showing a catalytic current onset at −2.29 V in 10 mM acetic acid, which shifts anodically by 85 mV −2.20 V upon increasing [CH_3_COOH] to 40 mM. Since only TFA among the two acids possesses sufficient acidity to protonate the formyl group, the pronounced differences observed suggest that the formyl group in [Ni­(OEP-CHO)] play an active role in protonation and shuttling of reducing equivalents during HER when TFA is used as the proton source.

**Figure 4:**
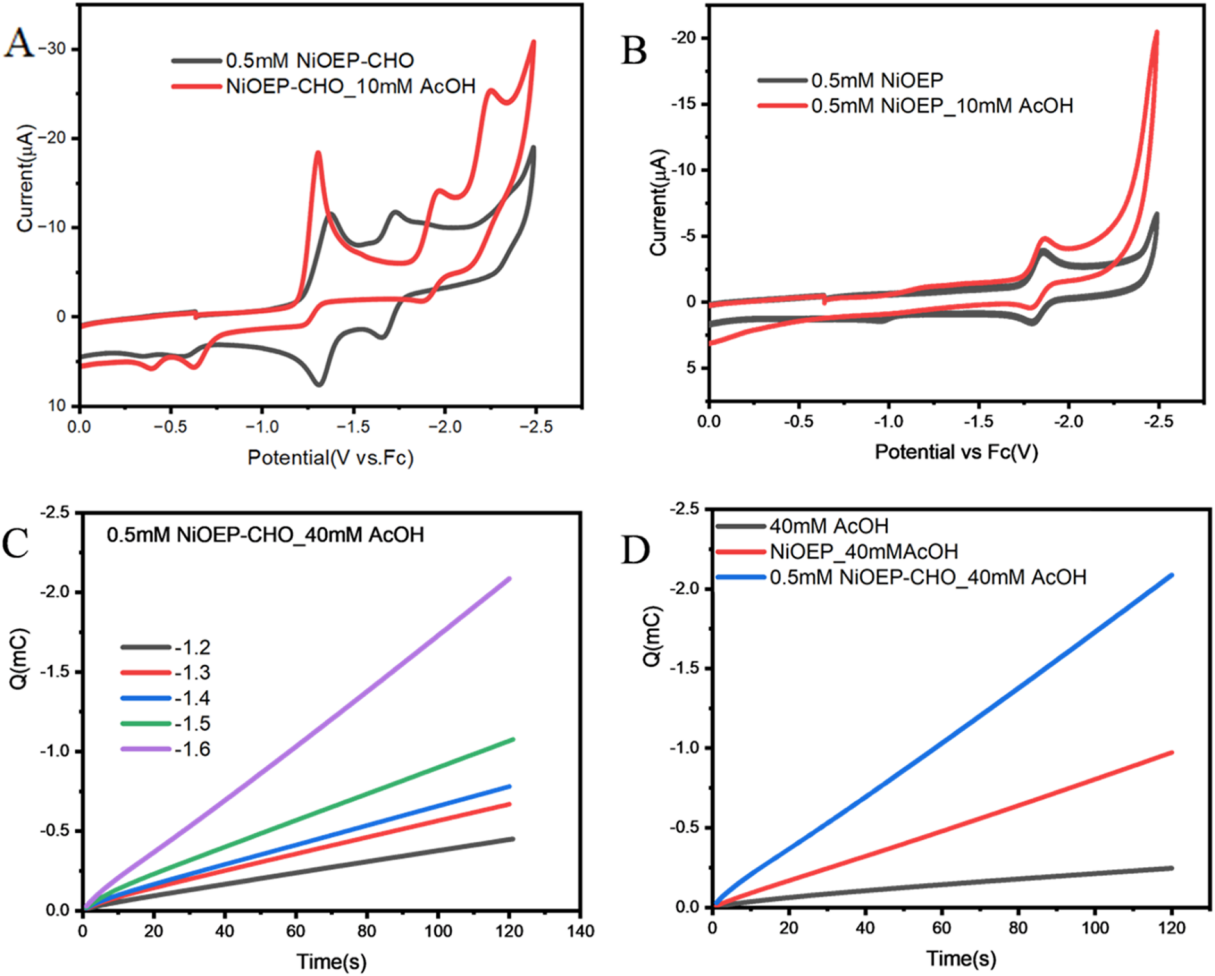
(A) Cyclic voltammograms of 0.5 mM [Ni­(OEP-CHO)] in the absence (black) and presence (red) of 10 mM acetic acid. (B) Cyclic voltammograms of 0.5 mM [Ni­(OEP)] in the absence (black) and presence (red) of 10 mM acetic acid. (C) Controlled potential electrolysis (CPE) of 0.5 mM [Ni­(OEP-CHO)] with 40 mM acetic acid at varying applied potentials. (D) Comparison of charge accumulation for [Ni­(OEP-CHO)] (blue), [Ni­(OEP)] (red), and 40 mM acetic acid alone (black) under an applied potential of −1.6 V.

Acetic acid, with a p*K*_a_ of 12.3 in DMF, is significantly weaker than TFA and induces only a modest increase in cathodic current for [Ni­(OEP-CHO)], accompanied by a slight positive shift (∼70 mV), without a distinct catalytic wave at the first reduction event. At potentials more negative than the second reduction couple observed in the absence of acid, new irreversible waves appear. These results suggest that, in the presence of acetic acid, [Ni­(OEP-CHO)] requires a two-step electrochemical reduction before initiating hydrogen evolution. Despite the lower acidity, gradual increases in charge accumulation were observed upon increasing the applied potential from −1.2 to −1.6 V in 40 mM acetic acid (Figure 4C). Fixed-potential electrolysis revealed that [Ni­(OEP-CHO)] outperforms [Ni­(OEP)] as an HER catalyst at −1.6 V over 120 s (Figure 4D). When the CPE experiment was extended to 3 h in 40 mM acetic acid, [Ni­(OEP-CHO)] accumulated 0.3212C of charge and produced 33.22 μL of H_2_, corresponding to a FE of 83%. In contrast, no hydrogen production was detected for [Ni­(OEP)], as −1.6 V was insufficient to drive catalysis. These results highlight the important role of the formyl group in [Ni­(OEP-CHO)], which acts as a redox mediator in the presence of acetic acid. Under strongly acidic TFA, the formyl group contributes both redox activity and proton-shuttling functionality, whereas in weaker acids like acetic acid, its contribution is limited to redox shuttling. In contrast, [Ni­(OEP)] displays no acid-dependent redox behavior in cyclic voltammograms, relying solely on the nickel center as both the redox-active and protonation site. The irreversible nature of the reduction waves observed for [Ni­(OEP-CHO)] in acetic acid suggests that the reduced species are unstable on the time scale of the voltametric experiment.

### Prominent Redox Processes and Electrocatalytic Pathway

The electron transfer processes and reaction pathways for electrocatalytic H_2_ generation by [Ni­(OEP-CHO)] were elucidated through cyclic voltammetry (CV), UV–vis spectroelectrochemistry, and DFT calculations. In the presence of TFA as proton sources, the first reduction wave of [Ni­(OEP-CHO)] undergoes a slight positive shift and increased current intensity but does not exhibit catalytic behavior. In contrast, the second reduction wave becomes catalytically active and maintains a nearly constant onset potential with increasing TFA concentration, consistent with an ECEC mechanism, where “E” denotes an electrochemical step and “C” denotes a chemical step.^31^ Controlled potential electrolysis (CPE) at the [Ni­(OEP-CHO)]^0/‑^ potential yielded no detectable hydrogen, indicating the absence of a bimetallic HER pathway involving [Ni­(H)­(OEP-CHO)]^−^ as the active species (Figure S12C). Thus, further reduction to form [Ni­(H)­(OEP-CHO)]^2–^ is required, which can release H_2_ through reaction with a hydronium source.

In the case of AcOH, the catalytic current appears well beyond the second reduction wave and shifts anodically with increasing acid concentration (Figure S3). This behavior suggests that hydrogen evolution in AcOH proceeds via a homolytic step, completing an EECC* reaction loop.^31^ As shown in Figure S5A, neutral [Ni­(OEP-CHO)] displays a split Soret band at 402 and 424 nm, and a broad Q-band spanning 600–700 nm. Upon application of −1.35 V vs Fc/Fc^+^, slightly beyond the first reduction potential, a weak band emerges near 275 nm along with a modest decrease in Soret band intensity (Figure 5A), indicating minor perturbation of the porphyrinic π–π* transitions. At a more negative potential (−1.71 V vs Fc/Fc^+^) in the absence of TFA, more pronounced spectral changes are observed (Figure 5B), including a significant drop in Soret band intensity, development of a shoulder at ∼468 nm, uniform attenuation of the Q bands, and increased intensity of the ∼275 nm band. These changes are consistent with metal-centered reduction processes reported previously.^3,12,24,32^

**Figure 5:**
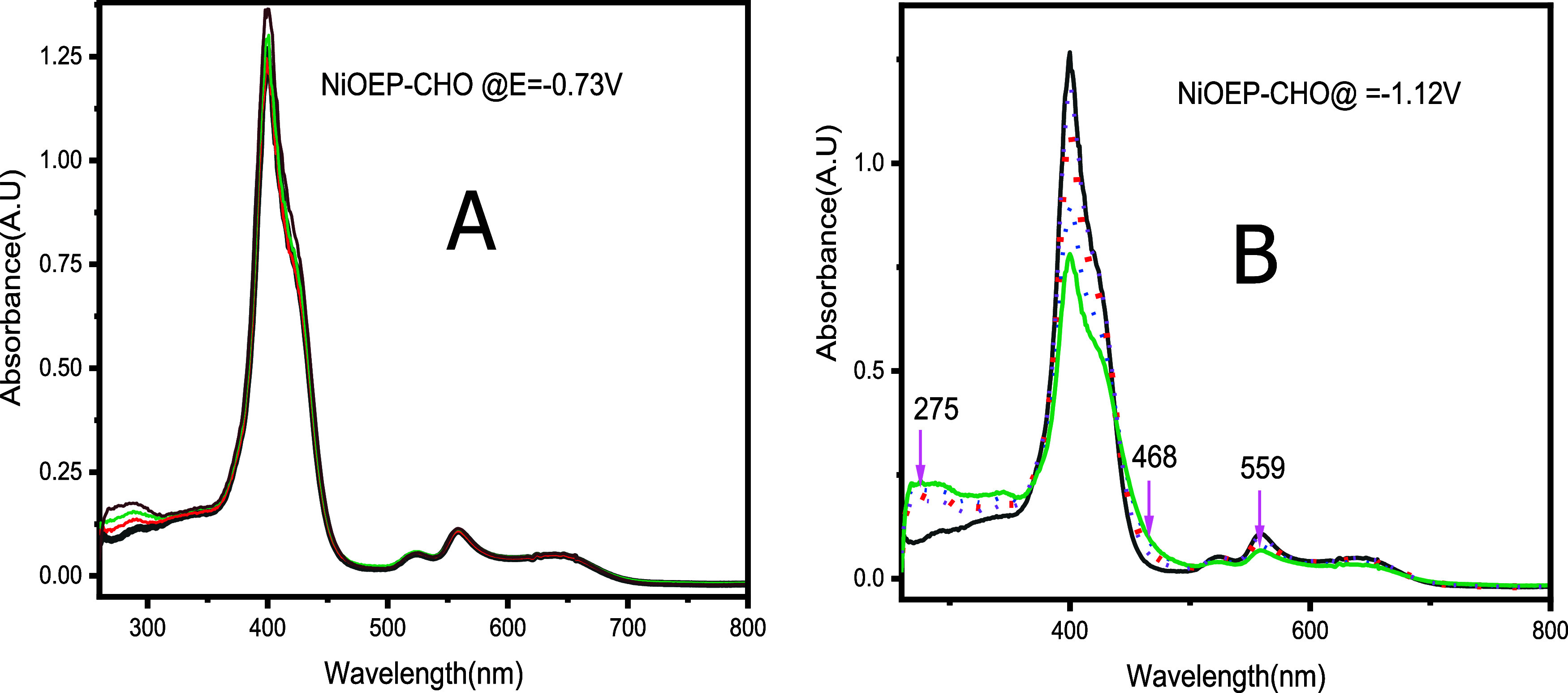
(A) UV–vis spectral changes of [Ni­(OEP-CHO)] upon electrochemical reduction at −0.73 V vs Ag/AgCl (−1.35 V vs Fc/Fc^+^). (B) UV–vis spectral changes at −1.12 V vs Ag/AgCl (−1.71 V vs Fc/Fc^+^).

The first reduction equivalent in [Ni­(OEP-CHO)] may be localized either on a metal-centered or a formyl porphyrin-based orbital. While one-electron reduction at the Ni­(II) center is generally more favorable and occurs at a higher potential than aldehyde reduction, conjugation between the porphyrinic π-system and the carbonyl moiety in [Ni­(OEP-CHO)] likely lowers the energy barrier for one-electron reduction at the formyl group as the initial redox site. The absence of a broad band in the near-IR region during UV–vis spectroelectrochemical analysis following one-electro reduction of [Ni­(OEP-CHO)] indicates the lack of a long-lived π-anion species, which would typically give rise to a phlorin-like intermediate upon protonation.^33^

Theoretical calculations for [Ni­(OEP-CHO)] revealed the charge density distributions of four representative molecular orbitals, LUMO+1, LUMO, HOMO, and HOMO – 1, in line with the conventional four-orbital model. (Figure 6). The two frontier orbitals—LUMO and LUMO + 1—are nearly degenerate (energy difference: 0.057 eV), while the energy gap between HOMO and HOMO – 1 is slightly larger at 0.230 eV. The overall HOMO–LUMO gap is calculated to be 3.189 eV. Charge density maps of the neutral [Ni­(OEP-CHO)] complex reveal significant hybridization between the formyl group orbitals and the porphyrinic π-system in the LUMO, indicating strong electronic communication between these fragments. Although the LUMO represents the orbital into which the incoming electron will be added upon reduction, it does not necessarily predict the final electron distribution in the reduced species. Nevertheless, the minimal contribution from metal d-orbitals in this frontier orbital supports the conclusion that the redox-active formyl group plays a key role in mediating electron transfer within the ligand framework. This interpretation is further corroborated by IR spectroelectrochemical measurements, which reveal a new band at 1606 cm^–1^ at −0.73 V vs Ag wire, suggesting a decrease in carbonyl bond order upon reduction (Figure S12a,b).

**Figure 6:**
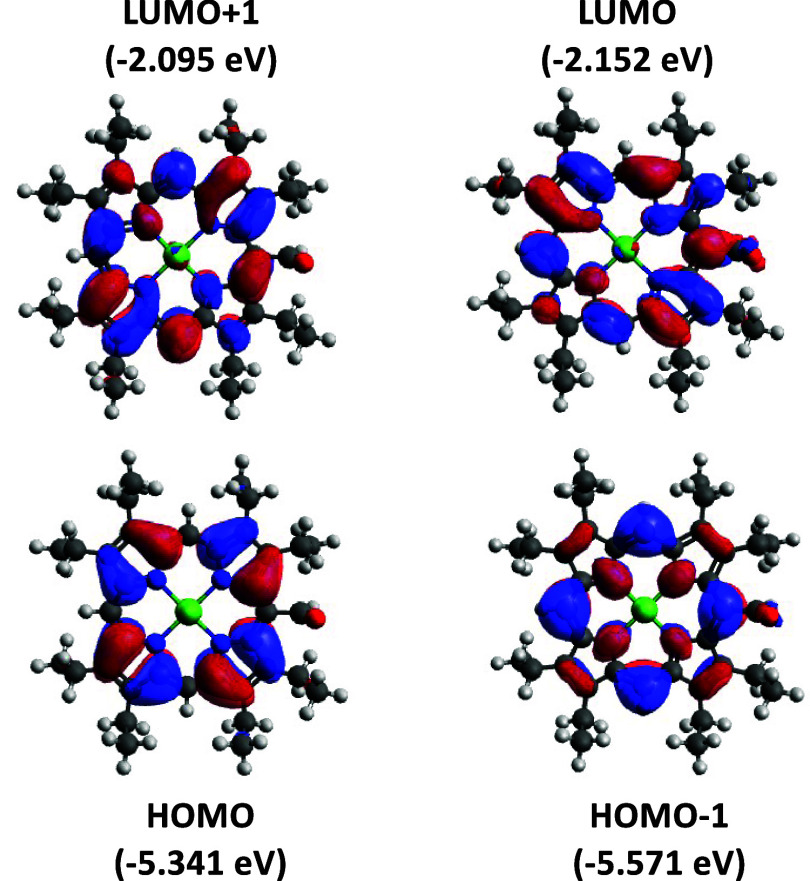
Molecular orbital distributions and relative energies of the frontier orbitals, including the HOMO, HOMO – 1, LUMO, and LUMO + 1, in [Ni­(OEP-CHO)]. The charge density maps illustrate the spatial distribution of electron density within these molecular orbitals.

Theoretical evaluation on the potential protonation sites in [Ni­(OEP-CHO)] indicates that protonation at the formyl group is 4.52 eV more favorable than at the Ni­(II) center, supporting the hypothesis that, in the presence of TFA, protonation preferentially occurs at the formyl moiety. Experimental HER activity, including current enhancement and hydrogen evolution with [Ni­(OEP)] as the catalyst (onset potential: −1.90 V), suggests that the nickel center remains a viable site for catalysis. For [Ni­(OEP-CHO)], following one-electron reduction and protonation, the resulting low-spin Ni­(III)-hydride species is calculated to be 29.96 kcal/mol more stable than its high-spin analog. Moreover, the low-spin hydride [H^–^–Ni­(III)­(OEP-CHO)] is 97.94 kcal/mol more stable than the initial protonated formyl intermediate. These results suggest that the hybridized conjugation between the formyl group and the porphyrinic π-system, along with its proximity to the nickel center, facilitates efficient proton and electron transfer to form a relatively stable Ni­(III)-hydride. Subsequent addition of a reducing equivalent and proton—likely aided by the formyl group—completes the hydrogen evolution cycle (Figure 7). The lower onset potential of −1.422 V (vs Fc/Fc^+^) for [Ni­(OEP-CHO)] in the presence of TFA, which closely matches its first reduction wave, supports the feasibility of an ECEC mechanism. Furthermore, the higher overpotentials observed for [Ni­(OEP)] in TFA and for [Ni­(OEP-CHO)] in acetic acid suggest that, in these systems, reduction precedes protonation—consistent with an EECC pathway.

**Figure 7:**
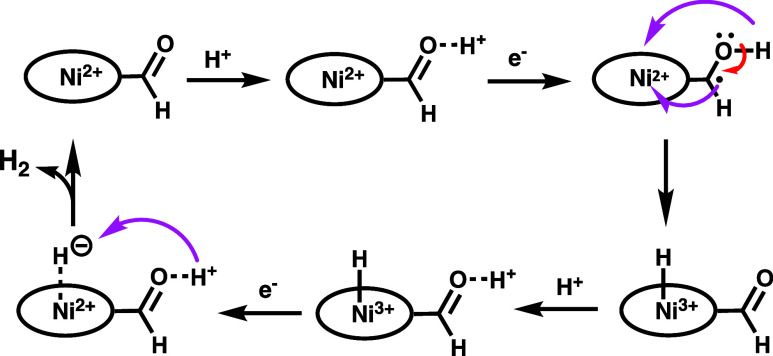
Schematic illustration of proposed HER pathway of [Ni­(OEP-CHO)] in the presence of TFA.

## Conclusions

This study demonstrates a significant enhancement in electrocatalytic hydrogen evolution (HER) via facial postsynthetic *meso*-functionalization of nickel­(II) octaethylporphyrin with a formyl group. Cyclic voltammetry reveals an additional anodically shifted redox couple, indicating the dual functionality of the formyl substituent. The formyl group acts both as a redox-active site and as a proton-shuttling relay, thereby lowering the energy barrier for HER. This dual role enables a turnover frequency of 0.54 ± 0.04 h^–1^ and a Faradaic efficiency of (96.5 ± 3.8)% at an overpotential of 128 mV in the presence of trifluoroacetic acid (TFA).

Cyclic voltammetry, spectroelectrochemical data, and DFT calculations collectively support a ligand-assisted mechanism, underscoring the critical role of the formyl group in facilitating both electron and proton transfer. These findings demonstrate that strategic *meso*-functionalization with a redox-active ligand can overcome the limitations of traditional electron-donating or -withdrawing substituents. This approach provides a powerful design principle for developing next-generation molecular catalysts for energy-related applications, including hydrogen production and other proton-coupled transformations. Future work may explore the incorporation of alternative functional groups to further tune catalytic performance and expand the scope of sustainable energy conversion strategies.

## Supplementary Material



## Notes

Tables of electrochemical and catalysis data, UV–visible, IR, and NMR spectra, and crystallographic atomic labeling information (PDF)
