# Supplementary material for: Meso-Formyl Functionalization Enhances Electrocatalytic Hydrogen Evolution Activity of Nickel(II) Octaethylporphyrin
Source: Inorg Chem. 2025 May 27;64(22):10792–800. doi: 10.1021/acs.inorgchem.5c00498 (PMC12152955; doi:10.1021/acs.inorgchem.5c00498)
Supplement: Supplementary file 1 [file ic5c00498_si_001.pdf]

Supplementary Information

Meso-Formyl Functionalization Enhances Electrocatalytic Hydrogen Evolution Activity of Nickel(II) Octaethylporphyrin

Tilahun Wubalem Tsega,[a,b,c,d] Danicah T. Agub,[e] Susan D. Arco,[e] Chi-Kwong Chang,[a] Chen-Hsiung Hung*[a,f]

[a]Institute of Chemistry, Academia Sinica, Nankang, Taipei 115201, Taiwan

[b]Sustainable Chemical Science and Technology Program, Taiwan International Graduate Program, Academia Sinica, Nankang, Taipei 115201, Taiwan

[c]Department of Applied Chemistry, National Yang Ming Chiao Tung University, Hsinchu 30010, Taiwan

[d]Department of Chemistry, College of Natural Sciences, Jimma University, P.O. Box 378, Jimma 251, Ethiopia

[e]Institute of Chemistry, University of the Philippines Diliman, Quezon City, Philippines 1101

[f]Department of Medicinal and Applied Chemistry, Kaohsiung Medical University, Kaohsiung 807378, Taiwan

Correspondence

Dr. Chen-Hsiung Hung

Institute of Chemistry, Academia Sinica

128 Section 2, Academia Road, Nankang, Taipei 115201, Taiwan

E-mail: chhung@gate.sinica.edu.tw

Experimental S3

Supporting Tables for electrochemical and catalysis S4

Turnover frequency calculations S5

Supporting Figures: Electrochemical and catalysis properties S7

Spectroscopy Supporting information of NiOEP-CHO S10

Single Crystal image S10

UV-Vis, IR, and ^{1}H NMR Spectra S11

Experimental

General Equations for Electrochemical and Catalytic Parameters

$$i_p = 0.4463\ nFAC\left(\frac{nFvD}{RT}\right)^{\frac{1}{2}} \ldots\ldots (1)$$

$$\frac{i_c}{i_p} = \frac{n}{0.4463}\sqrt{\frac{RTk_{obs}}{Fv}} \ldots\ldots\ldots\ldots (2)$$

$$\%FE = \frac{z_{H2} \times 2\ F}{Q} \ldots\ldots\ldots\ldots\ldots\ldots\ldots (3)$$

$$\eta = E^{o}{}_{HA/H_2} - E_{cat/2} \ldots\ldots\ldots (4)$$

$$E^{o}{}_{HA/H_2} = E^{o}{}_{H^+/H2} - \frac{2.303RT}{F} pKa \ldots. (5)$$

$$TOF = \frac{\text{moles of product}}{\text{moles of catalyst active form} * \text{time}} \ldots\ldots\ldots (6)$$

$$[Active\ form] = \frac{Charge\ transferred}{F} \ldots\ldots\ldots (7)$$

Where:

- *n* is the number of electrons transferred
- *F* is the Faraday constant (96,485 $C \cdot mol^{-1}$)
- *A* is the electrode surface area (cm^2)
- *C* is the catalyst concentration ($mol \cdot cm^{-3}$)
- *v* is the scan rate ($V \cdot s^{-1}$)
- *D* is the diffusion coefficient ($cm^2 \cdot s^{-1}$)
- *R* is the universal gas constant (8.314 $J \cdot mol^{-1} \cdot K^{-1}$)
- *T* is the temperature (K)
- k_{obs} is the observed rate constant
- z_{H2} is the number of moles of H_2 (determined from GC analysis)
- *Q* is the total charge passed during electrolysis (C)
- η is the overpotential
- pK_a is the acid dissociation constant
- $E_{cat/2}$ is the half-wave potential of the catalyst
- $E^{o}{}_{HA/H_2}$ is the standard potential for the H^+/H_2 couple

Supporting Tables for electrochemical and catalysis

**Table 1.** Redox half-wave potentials and peak-to-peak separations between anodic and cathodic waves			
Parameter	[Ni(OEP-CHO)]		[Ni(OEP)]
E (V vs Fc^+/Fc)	-1.348 (II/I)	-1.709 (I/0)	-1.824 (II/I)
i_p (μA)	-9.32	-9.94	-3.652
ΔE mV	63	74	59
i_{pa}/ip_c	1.252	3.384	3.0173

Where i_p is reversible cathodic current as no acid present, the numeric in the bracket denotes peak separation between reduction and oxidation potential.

**Table 2.** Catalytic responses at varying the concentrations of TFA						
[Ni(OEP-CHO)]				[Ni(OEP)]		
[TFA], mM	ipl	ipl/ip(II/I)	ipl/ip(I/0)	[TFA], mM	ipl	ipl/ip(II/I)
0	-9.32E-06	1	1	0	-3.65E-06	1
5	-2.49E-04	26.71389	25.21399	5.5	-2.04E-04	55.80394
15	-7.87E-04	84.48253	79.22941	15	-5.62E-04	154.02865
20.5	-7.37E-04	79.06613	74.10421	20.5	-7.98E-04	218.54549

**Table 3.** Parameters of catalytic efficiency at 20.5 mM of TFA		
Potential	Catalyst	
	[Ni(OEP-CHO)]	[Ni(OEP)]
Onset E(V) vs Fc^+/Fc	-1.608	-1.855
$E_{-Cat}/2$(V) vs Fc^+/Fc	-2.028	-2.089
E_{-peak}(V) vs Fc^+/Fc	-2.458	-2.364V
Peak area (H_2 μmol)	278.604±20.419 (8.04±0.61)	27.795(0.75)

Charges passed in 3 hrs (C)	1.606±0.080	0.1853

**Table 4.** Selected crystallographic parameters for [Ni(OEP-CHO)]	
Code	i19593
Empirical formula	$C_{37}H_{44}N_4NiO$
Formula weight	619.47
Temperature	100.0(2) K
Wavelength	1.54178 Å
Crystal system	Triclinic
Space group	P-1
Unit cell dimensions	a = 4.8095(3) Å, α= 68.001(3)°<br>b = 12.8139(9) Å, □= 89.532(3)°<br>c = 13.4104(9) Å, γ = 84.498(3)°
Volume	762.41(9) $Å^3$
Z	1
Density (calculated)	1.349 Mg/m^3
Absorption coefficient	1.195 $mm^{-1}Mg/m^3$
Crystal size	0.513 x 0.079 x 0.033 mm^3
Theta range for data collection	3.556 to 68.614°

Calculations of turnover frequency

The turnover frequency (TOF) was estimated based on triplicate gas chromatography (GC) measurements, using a pre-calibrated curve constructed from standard hydrogen. The amount of hydrogen produced (in volume) was determined by correlating each GC peak area to the calibration curve, and subsequently converted to moles using the ideal gas law, assuming 1 mol of gas occupies 24 L at room temperature and 1 atm pressure. TOF was then calculated by assuming that only the active form of the catalyst—residing within the reaction diffusion layer—participates in

the catalytic process. The concentration of the active catalyst species was determined by dividing the total charge passed by the Faraday constant.

Table 5. Turnover frequencies (TOFs) from triplicate 3 h CPE measurements using 0.5 mM catalyst in 10 mL DMF containing supporting electrolyte.

Sample ID	250517 S1	250517S2	250517S3	Mean	σ	μ±σ
Q	1.533	1.593	1.691	1.606	0.080	1.606±0.080
PA	287.95	283.40	324.70	298.68	22.648	278.604±20.419
H_2(μmol)	7.75	7.62	8.74	8.04E+00	0.609	8.04±0.61
TOF(h^{-1})	0.520	0.508	0.582	0.536	0.041	0.536 ± 0.041
FE%	97.51	92.33	99.69	96.511	3.777	96.511±3.777

$$\mathrm{TOF} = \frac{\text{moles of product}}{\text{moles of catalyst used} * \text{time of reaction}}$$

Supporting Figures: Electrochemical and catalysis properties

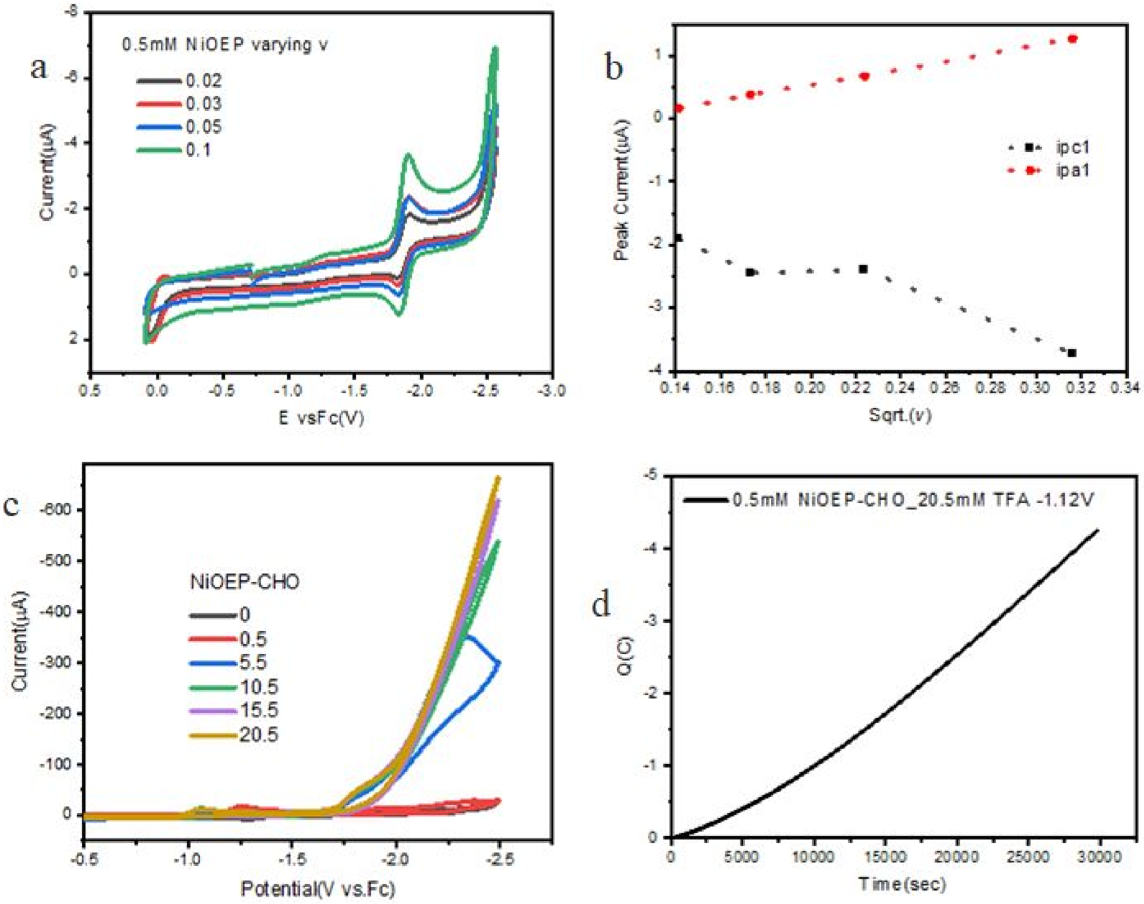

Figure S1.
(A) Cyclic voltammograms (CVs) of 0.5 mM [Ni(OEP)] recorded at increasing scan rates.
(B) Plot of peak current (i□) versus the square root of scan rate ($v^{1/2}$) for 0.5 mM [Ni(OEP-CHO)].
(C) CVs of [Ni(OEP-CHO)] recorded at varying concentrations of TFA.
(D) Extended controlled potential electrolysis (CPE) of 0.5 mM [Ni(OEP-CHO)] with 20.5 mM TFA at –1.12 V for 8 hours.

All experiments were performed in 0.1 M $[Bu_4N]PF_6$/DMF solution at room temperature under a nitrogen atmosphere. In the cyclic voltammograms, $i□_c$ and $i□_a$ represent the cathodic and anodic peak currents, respectively, while labels 1 and 2 correspond to the associated redox waves.

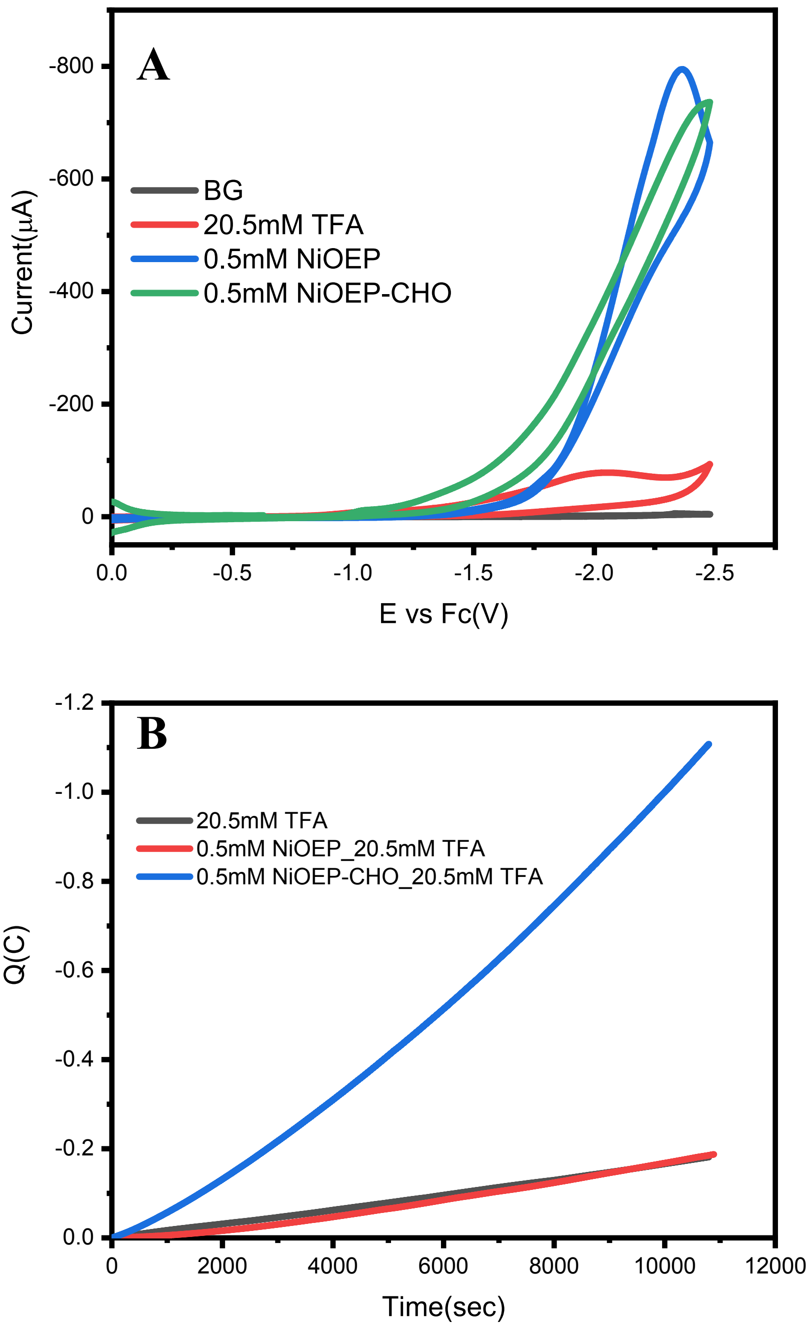

Figure S2. (A) Cyclic voltammograms (CVs) of 0.5 mM [Ni(OEP-CHO)] and [Ni(OEP)] recorded in the presence of 20.5 mM TFA at a scan rate of 100 mV·s^{-1} in 0.1 M $[Bu_4N]PF_6$/DMF solution. (B) Controlled potential electrolysis (CPE) of 0.5 mM [Ni(OEP-CHO)] and [Ni(OEP)] conducted at –1.12 V in 0.1 M TFA/0.1 M $[Bu_4N]PF_6$/DMF solution for 3 hours.

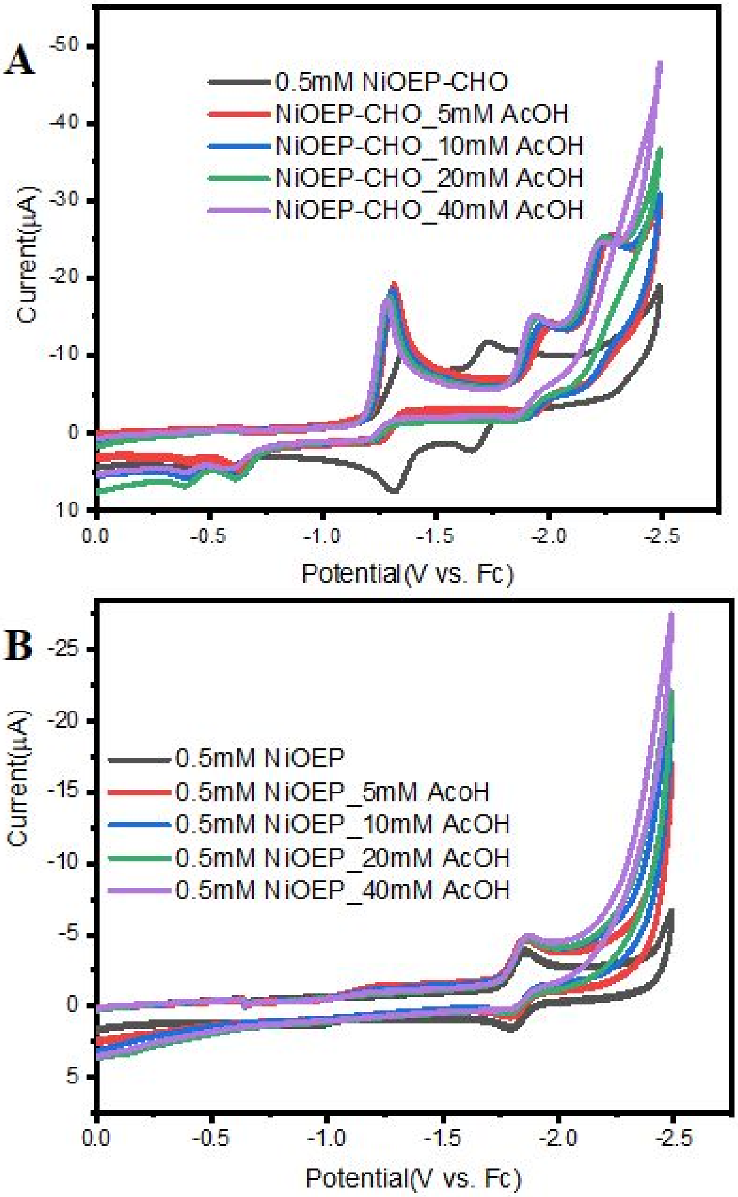

Figure S3. Cyclic voltammograms (CVs) recorded at 0.1 V·s^{-1} in 0.1 M $[Bu_4N]PF_6$/DMF with varying concentrations of AcOH: 0 mM (black), 5 mM (red), 10 mM (blue), 20 mM (green), and 40 mM (magenta).

(A) 0.5 mM [Ni(OEP-CHO)]

(B) 0.5 mM [Ni(OEP)]

Spectroscopy Supporting information of NiOEP-CHO

Single Crystal image

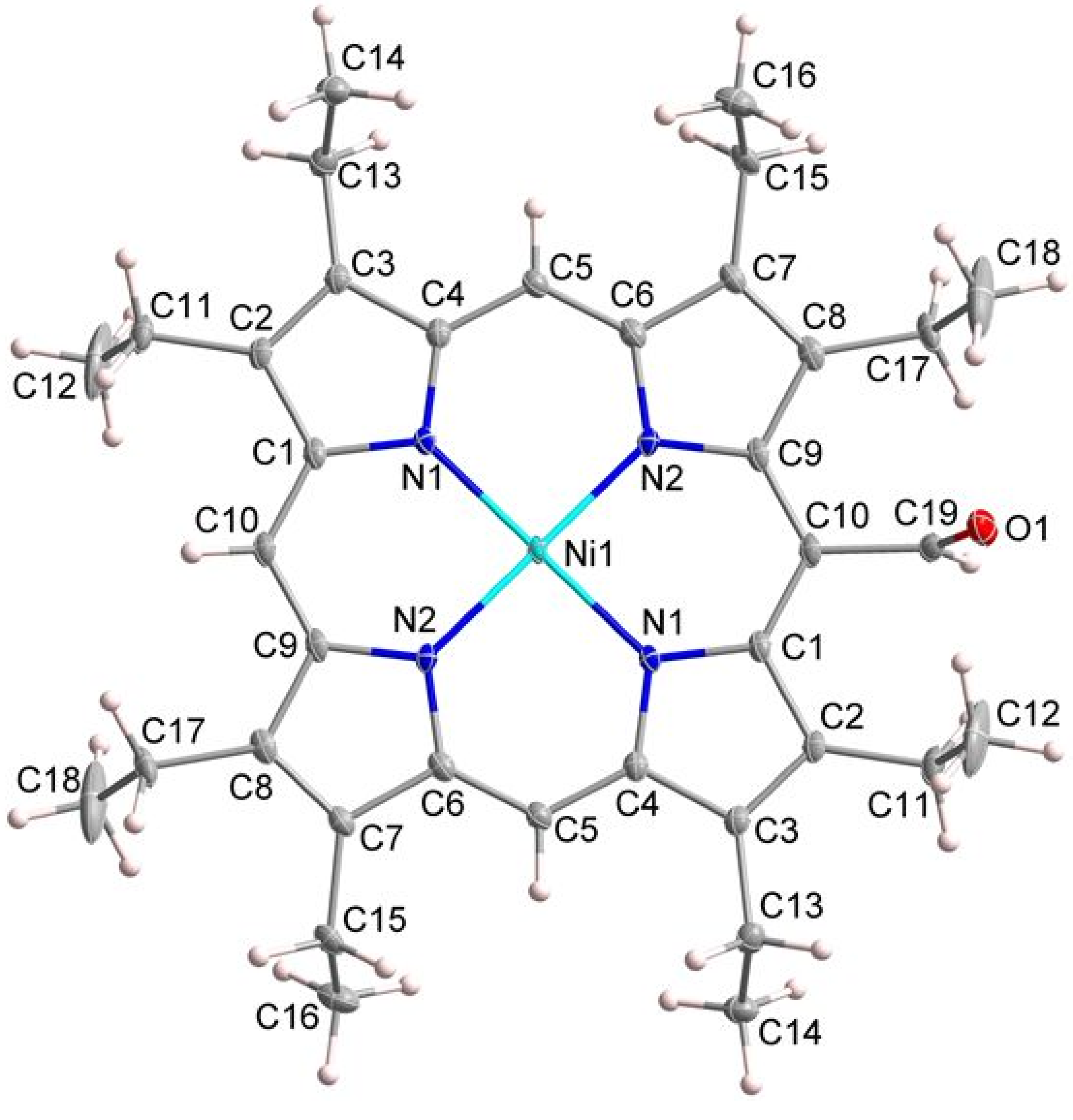

Figure S4. ORTEP representation of the single-crystal structure of [Ni(OEP-CHO)] with atomic labeling.

UV-Vis, IR, and ^{1}H NMR Spectra

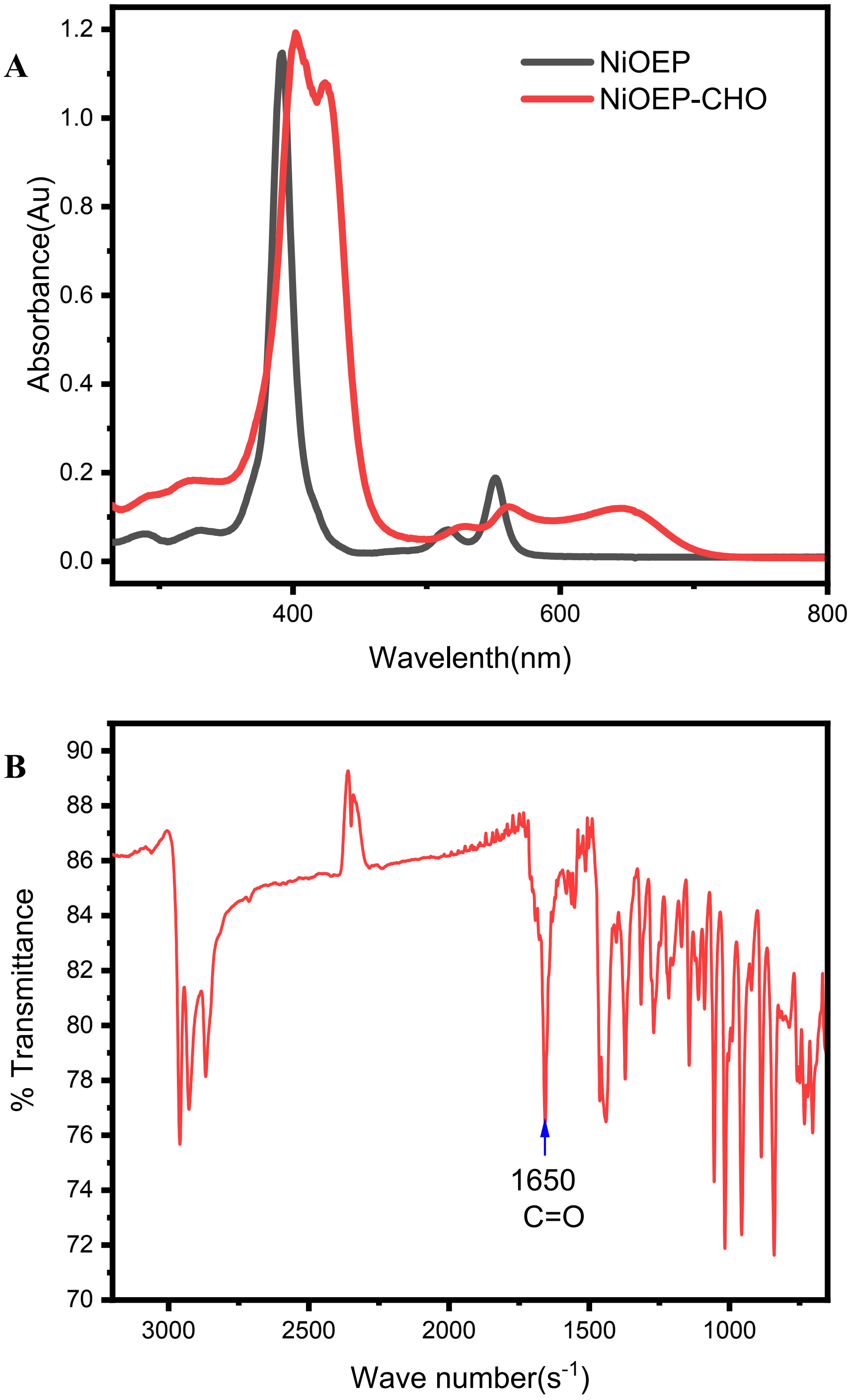

Figure S5. (A) UV–Vis spectra of [Ni(OEP-CHO)] (red trace) and [Ni(OEP)] (black trace) in dichloromethane. Compared to [Ni(OEP)], [Ni(OEP-CHO)] exhibits a slightly red-shifted and shouldered Soret band, along with a broadened Q band.
(B) ATR-FTIR spectrum of [Ni(OEP-CHO)].

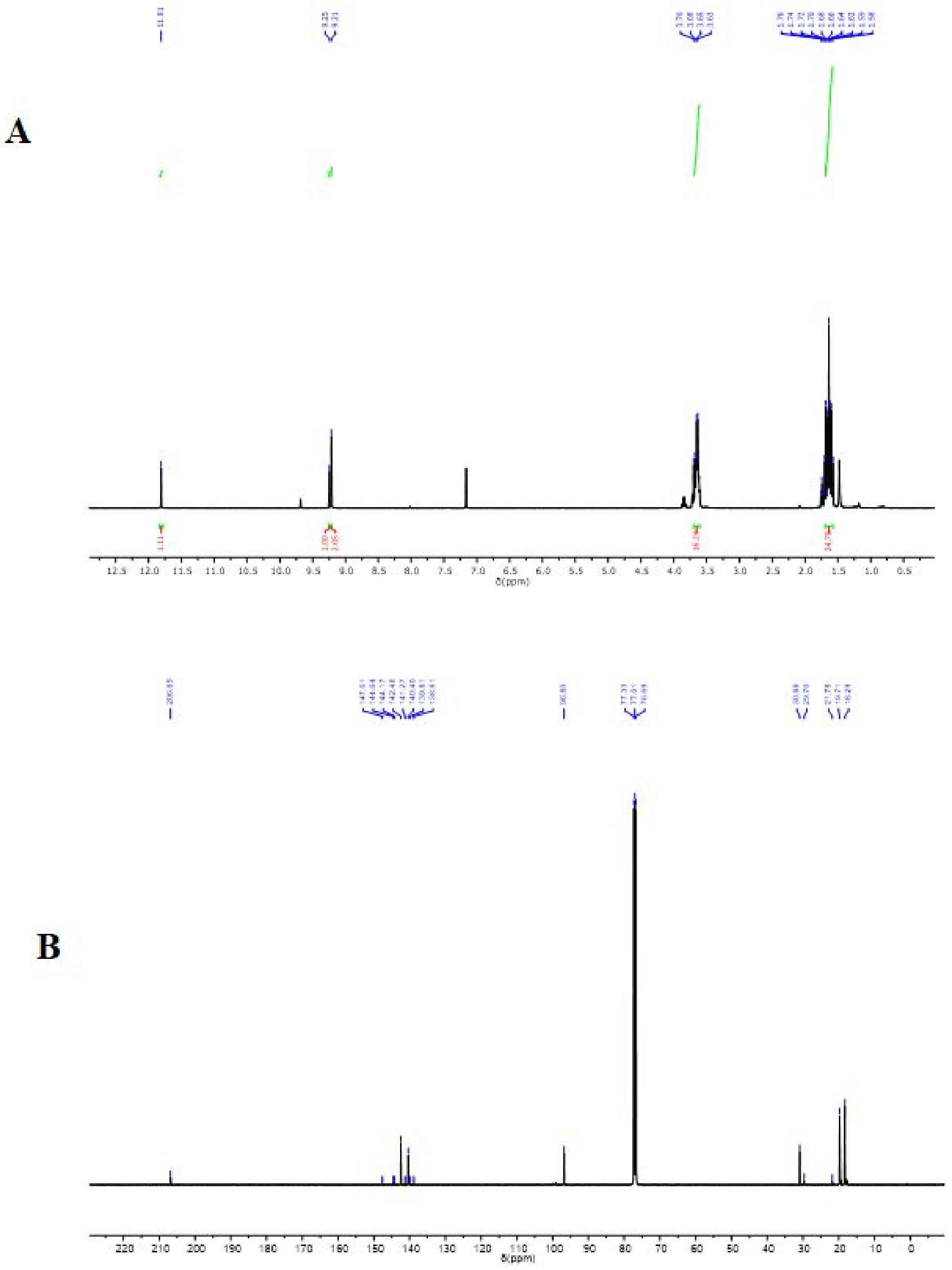

Figure S6.

Full NMR spectra of [Ni(OEP-CHO)] recorded in $CDCl_3$: (A) ^{1}H NMR spectrum; (B) $^{13}C\{^{1}H\}$ NMR spectrum.

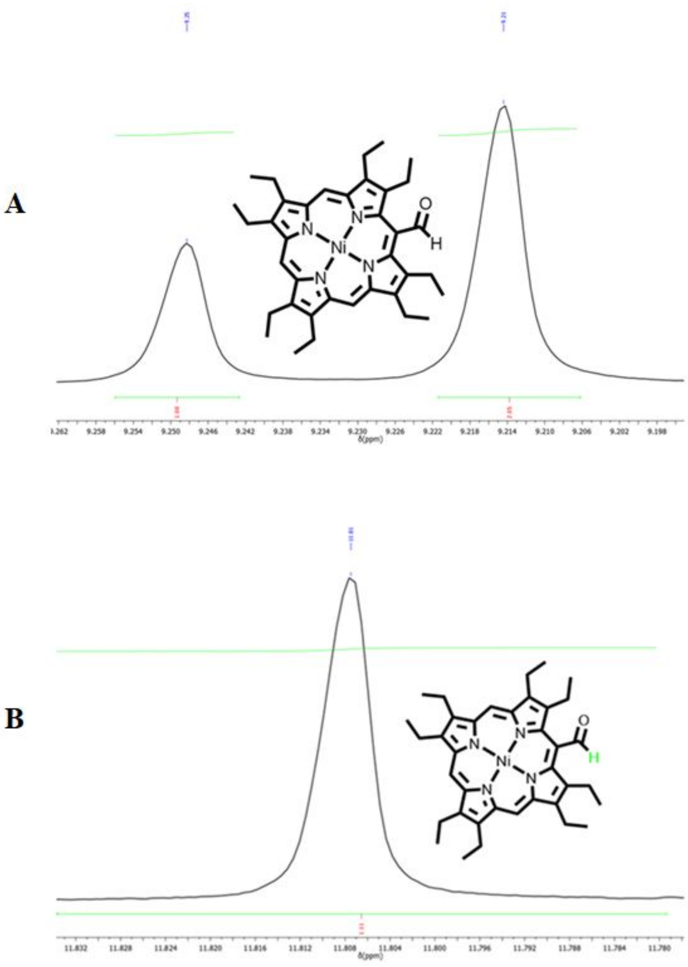

Figure S7.
^{1}H NMR spectra of [Ni(OEP-CHO)] highlighting the (A) aromatic (meso C=CH) region and (B) aliphatic (meso C–CH) region.

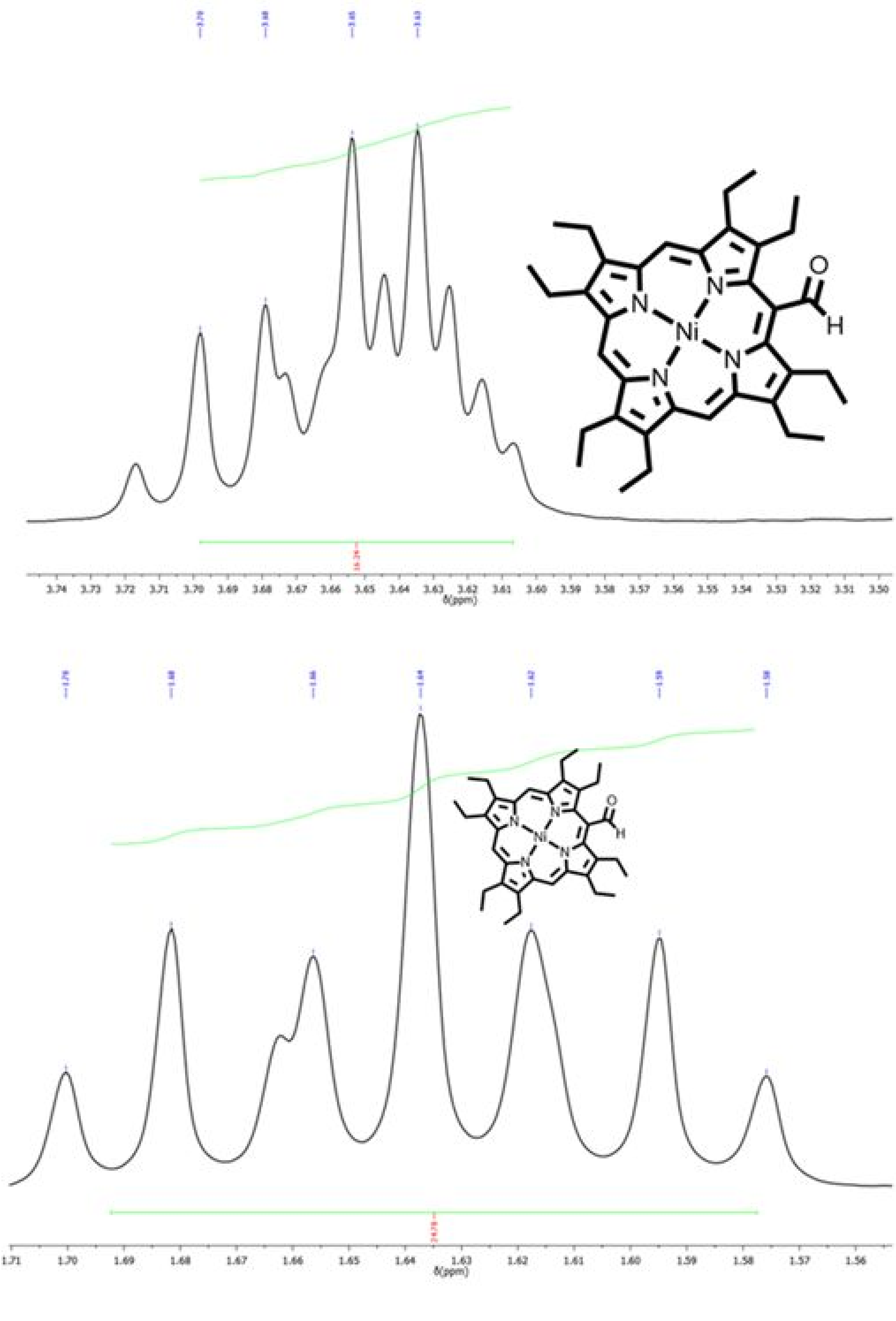

Figure S8. Aliphatic region of the ^{1}H NMR spectrum of [Ni(OEP-CHO)] recorded in $CDCl_3$.

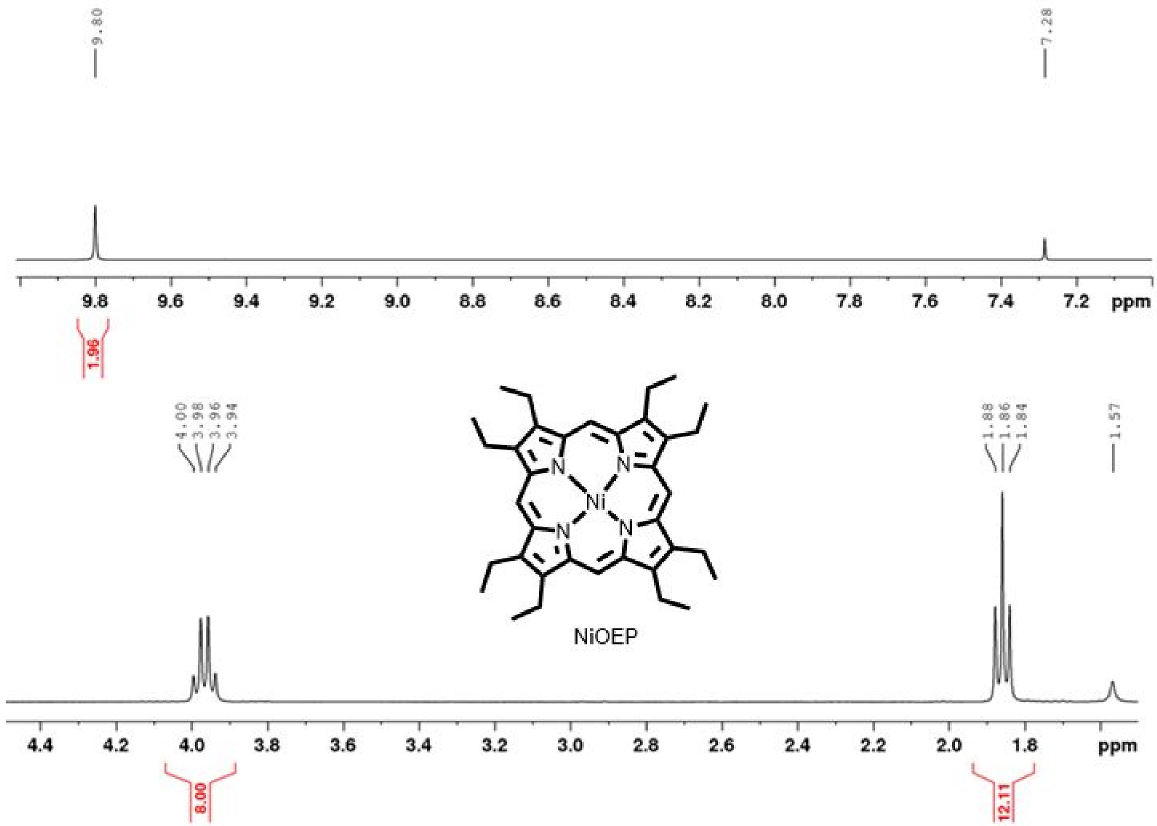

Figure S9. Full ^{1}H NMR spectrum of [Ni(OEP)] recorded in $CDCl_3$.

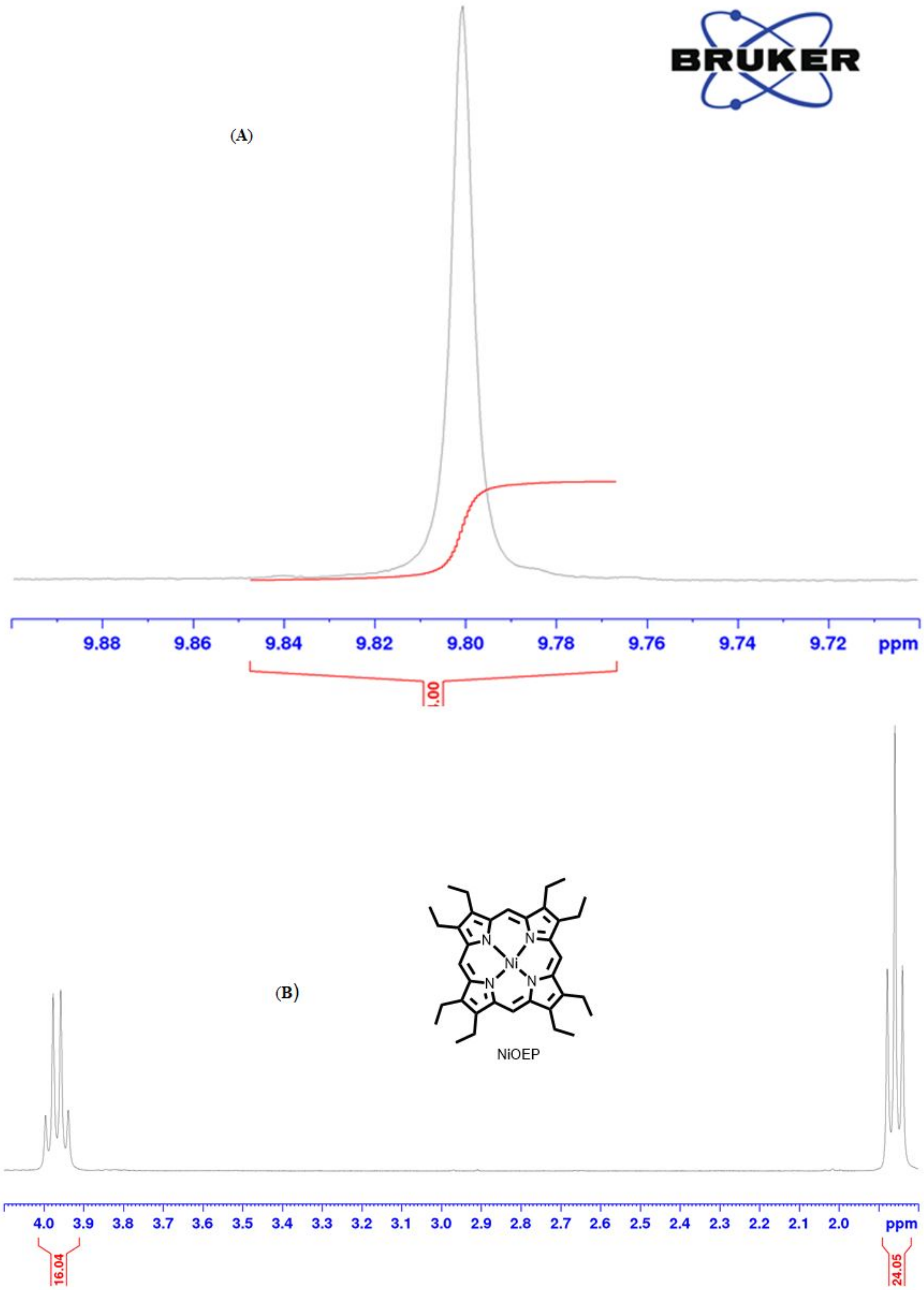

Figure S10.

^{1}H NMR spectrum of [Ni(OEP)] recorded in $CDCl_3$, highlighting (A) the aromatic region and (B) the aliphatic region.

NiOEP-CHO (HR-ESI)

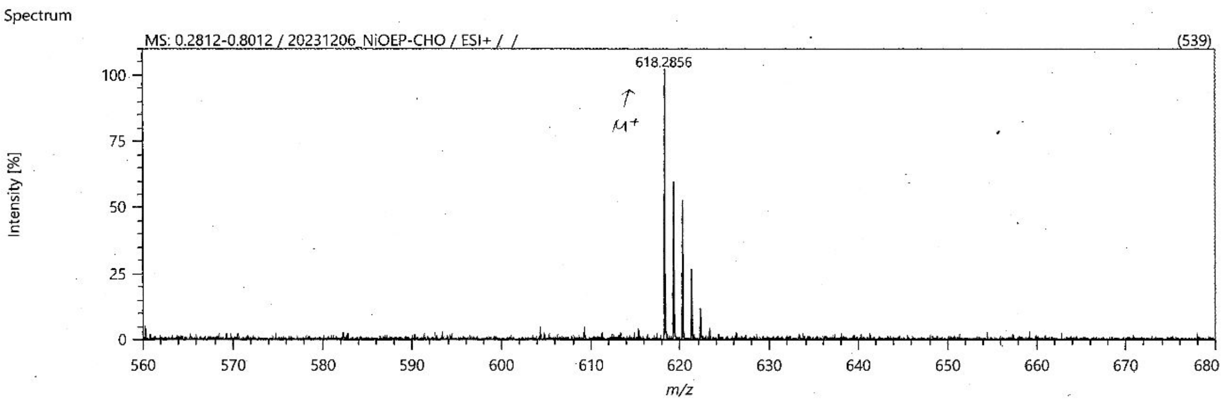

Elemental Composition

Parameters	
Tolerance:	±5.00 ppm
Electron:	Odd/Even
Charge:	+1
DBE:	-99.0 - 999.0

Elements Set 2:

Symbol	C	H	O	N	Ni
Min	0	0	1	4	1
Max	100	1000	1	4	1

Results

Mass	Formula	Calculated Mass	Mass Difference [mDa]	Mass Difference [ppm]	DBE
618.28556	C37 H44 N4 O Ni	618.28631	-0.75	-1.21	18.5

Figure S11. High-resolution electrospray ionization mass spectrum (HR-ESI-MS) of [Ni(OEP-CHO)].

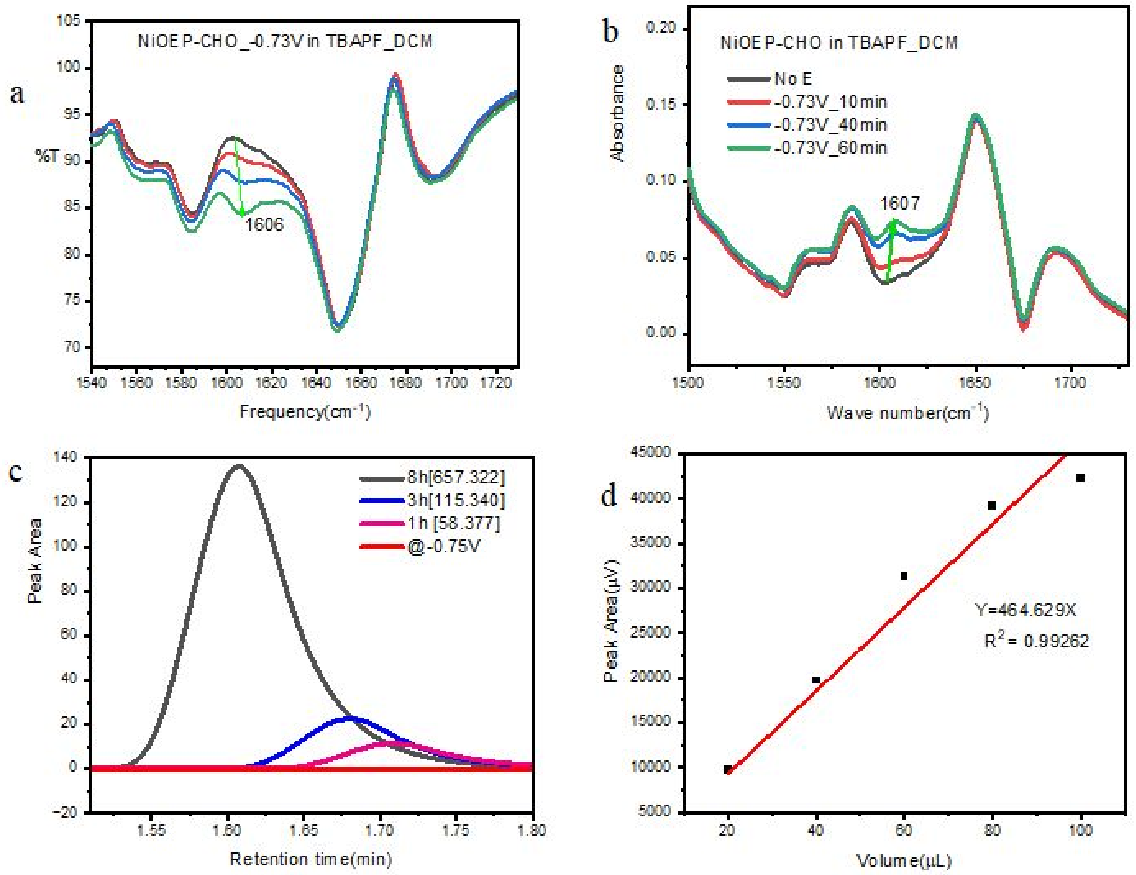

Figure S12.

(a) IR spectroelectrochemical (SEC) spectrum of [Ni(OEP-CHO)] in CH_2Cl_2 displayed as percent transmittance.

(b) IR-SEC spectrum of [Ni(OEP-CHO)] presented in absorbance mode.

(c) Gas chromatogram of H_2 generated from [Ni(OEP-CHO)] during electrolysis at –1.12 V.

(d) Calibration curve for H_2 quantification using standard hydrogen samples.

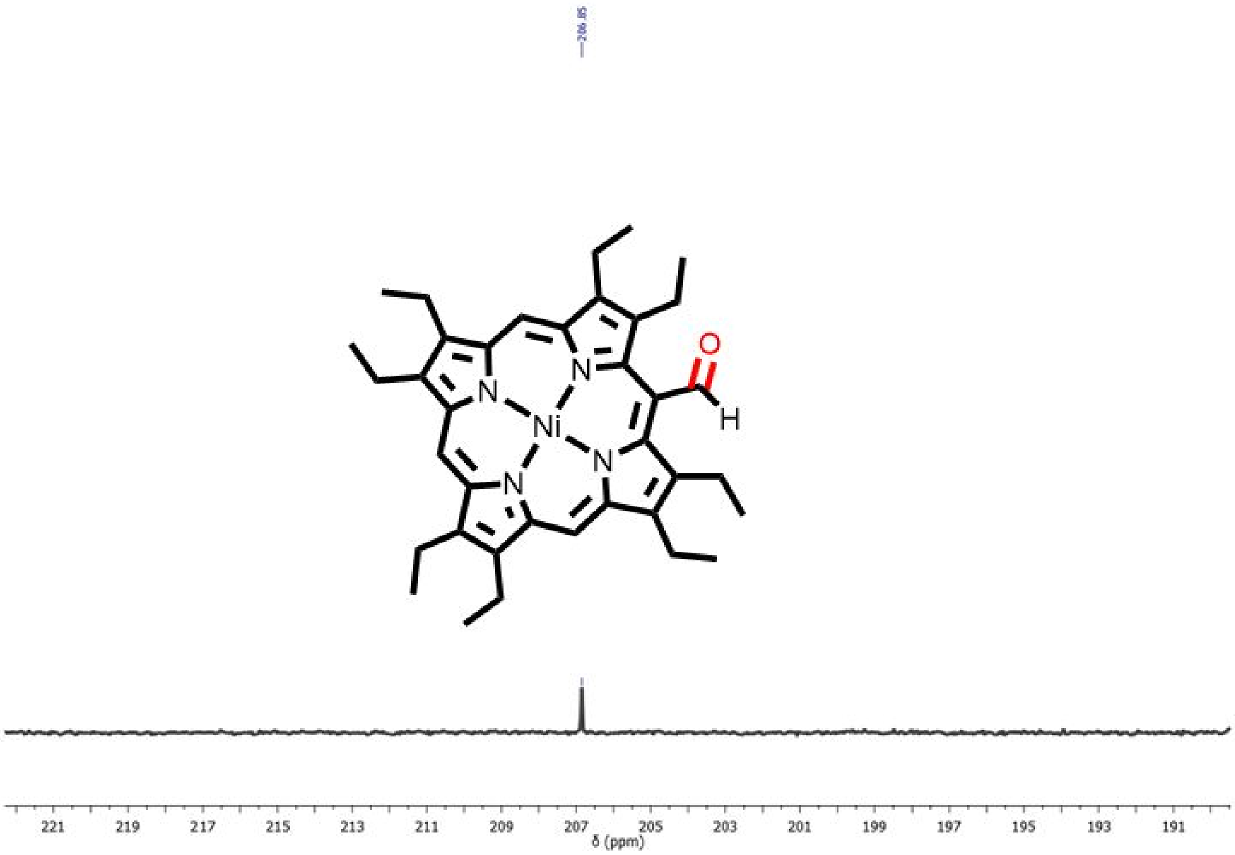

Figure S13. Aliphatic region of the $^{13}C\{^{1}H\}$ NMR spectrum of [Ni(OEP-CHO)].

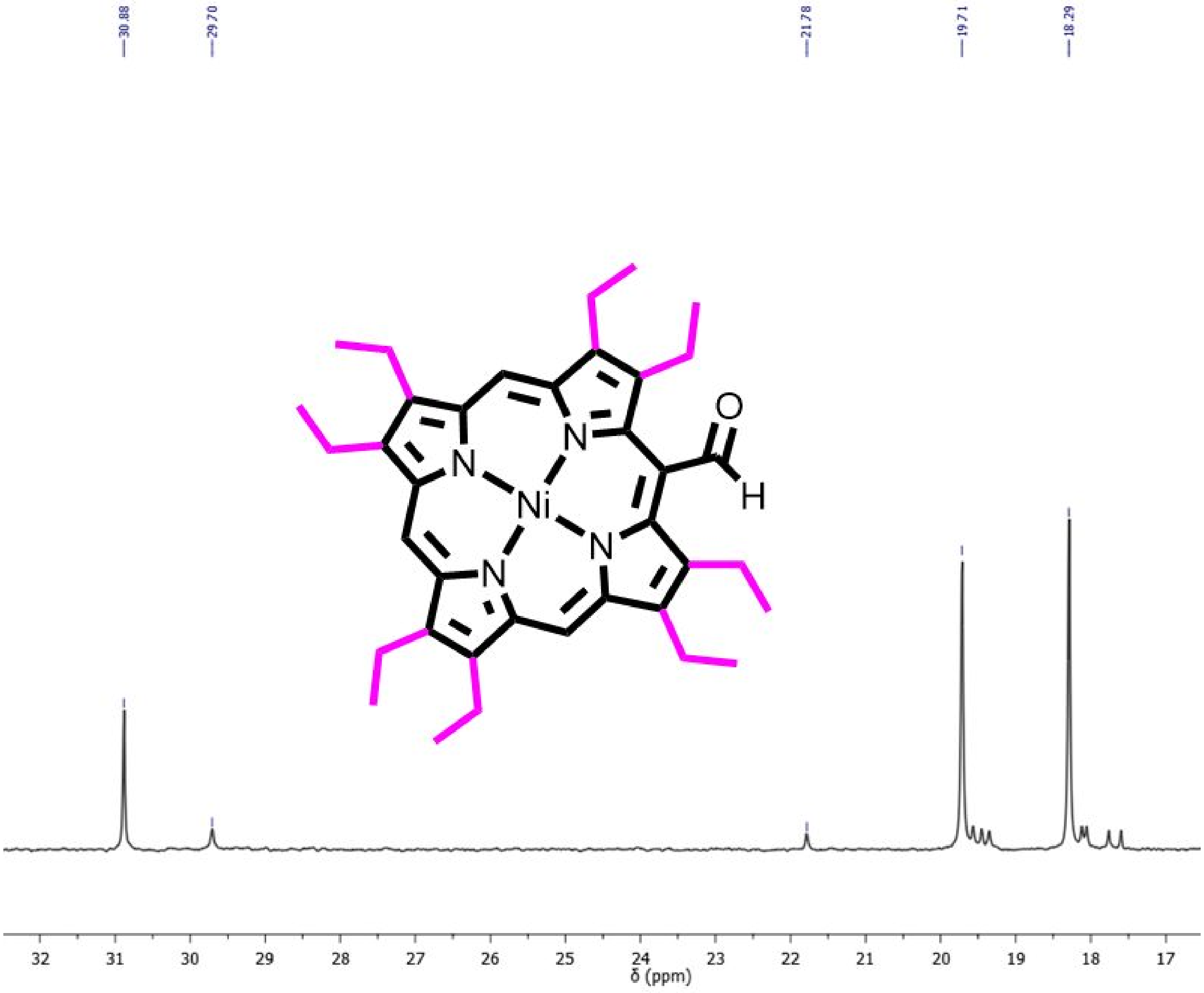

Figure S14. Aliphatic region of the $^{13}C\{^{1}H\}$ NMR spectrum of [Ni(OEP-CHO)].

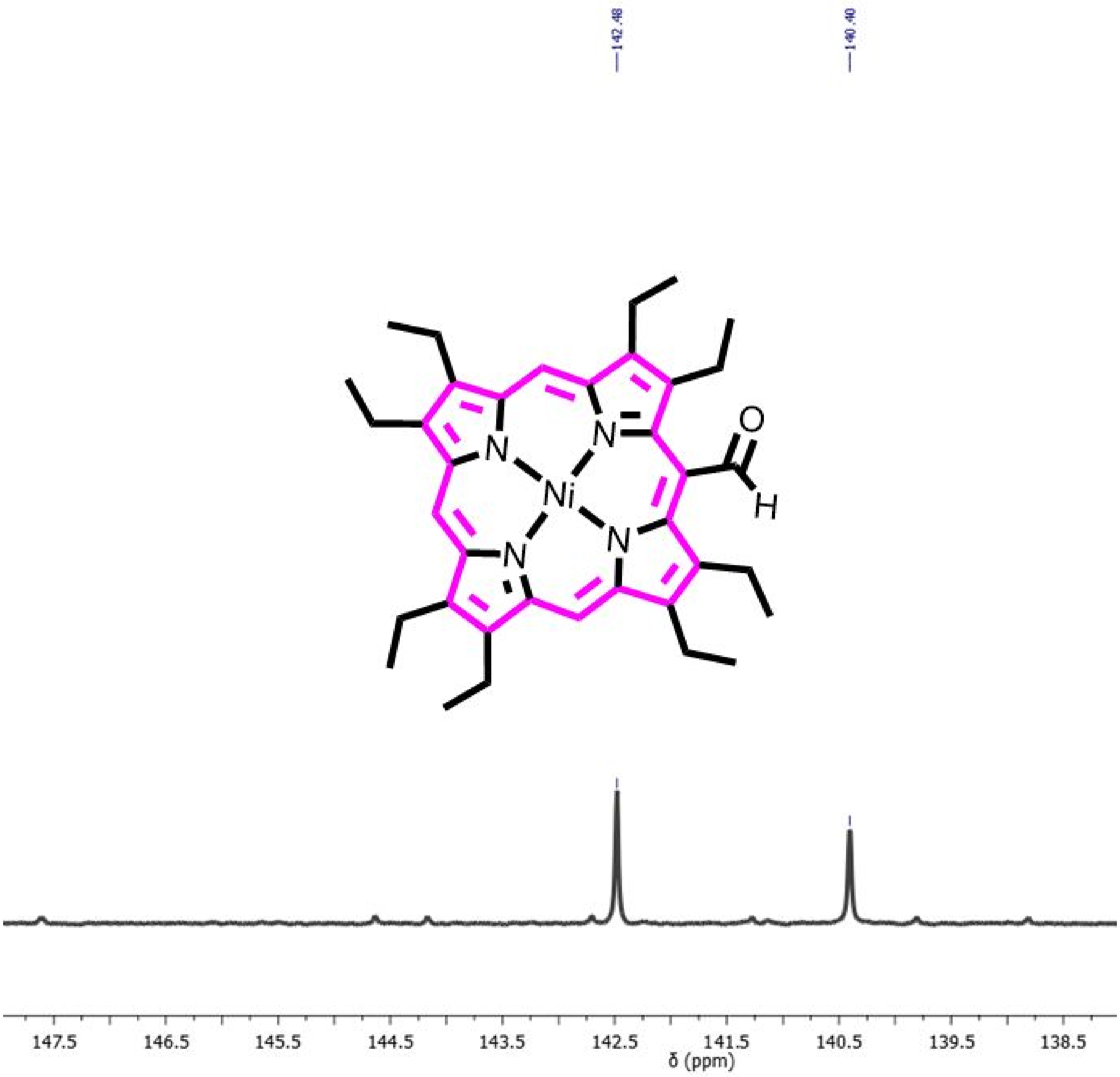

Figure S15. Aromatic region of the $^{13}C\{^{1}H\}$ NMR spectrum of [Ni(OEP-CHO)].
